# Intravenous infusions of mesenchymal stromal cells have cumulative beneficial effects in a porcine model of chronic ischaemic cardiomyopathy

**DOI:** 10.1093/cvr/cvae173

**Published:** 2024-08-20

**Authors:** Xian-Liang Tang, Marcin Wysoczynski, Anna M Gumpert, Mitesh Solanki, Yan Li, Wen-Jian Wu, Shirong Zheng, Halina Ruble, Hong Li, Heather Stowers, Shengnan Zheng, Qinghui Ou, Nida Tanveer, Jan Slezak, Dinesh K Kalra, Roberto Bolli

**Affiliations:** Institute of Molecular Cardiology, University of Louisville, 550 S Jackson Street, ACB Bldg, 3rd Floor, Louisville, KY 40202; Institute of Molecular Cardiology, University of Louisville, 550 S Jackson Street, ACB Bldg, 3rd Floor, Louisville, KY 40202; Institute of Molecular Cardiology, University of Louisville, 550 S Jackson Street, ACB Bldg, 3rd Floor, Louisville, KY 40202; Institute of Molecular Cardiology, University of Louisville, 550 S Jackson Street, ACB Bldg, 3rd Floor, Louisville, KY 40202; Institute of Molecular Cardiology, University of Louisville, 550 S Jackson Street, ACB Bldg, 3rd Floor, Louisville, KY 40202; Institute of Molecular Cardiology, University of Louisville, 550 S Jackson Street, ACB Bldg, 3rd Floor, Louisville, KY 40202; Institute of Molecular Cardiology, University of Louisville, 550 S Jackson Street, ACB Bldg, 3rd Floor, Louisville, KY 40202; Institute of Molecular Cardiology, University of Louisville, 550 S Jackson Street, ACB Bldg, 3rd Floor, Louisville, KY 40202; Institute of Molecular Cardiology, University of Louisville, 550 S Jackson Street, ACB Bldg, 3rd Floor, Louisville, KY 40202; Institute of Molecular Cardiology, University of Louisville, 550 S Jackson Street, ACB Bldg, 3rd Floor, Louisville, KY 40202; Institute of Molecular Cardiology, University of Louisville, 550 S Jackson Street, ACB Bldg, 3rd Floor, Louisville, KY 40202; Institute of Molecular Cardiology, University of Louisville, 550 S Jackson Street, ACB Bldg, 3rd Floor, Louisville, KY 40202; Institute of Molecular Cardiology, University of Louisville, 550 S Jackson Street, ACB Bldg, 3rd Floor, Louisville, KY 40202; Centre of Experimental Medicine, Institute for Heart Research, Bratislava, Slovakia; Institute of Molecular Cardiology, University of Louisville, 550 S Jackson Street, ACB Bldg, 3rd Floor, Louisville, KY 40202; Institute of Molecular Cardiology, University of Louisville, 550 S Jackson Street, ACB Bldg, 3rd Floor, Louisville, KY 40202

**Keywords:** Adult stem cells, Intravenous, Ischaemic cardiomyopathy, Myocardial infarction, Cardiac repair, Swine, Cell therapy, Fibrosis, Inflammation

## Abstract

**Aims:**

The development of cell therapy as a widely available clinical option for ischaemic cardiomyopathy is hindered by the invasive nature of current cell delivery methods. Furthermore, the rapid disappearance of cells after transplantation provides a cogent rationale for using repeated cell doses, which, however, has not been done thus far in clinical trials because it is not feasible with invasive approaches. The goal of this translational study was to test the therapeutic utility of the intravenous route for cell delivery.

**Methods and results:**

Pigs with chronic ischaemic cardiomyopathy induced by myocardial infarction received one or three intravenous doses of allogeneic bone marrow mesenchymal stromal cells (MSCs) or placebo 35 days apart. Rigour guidelines, including blinding and randomization, were strictly followed. A comprehensive assessment of left ventricular (LV) function was conducted with three independent methods (echocardiography, magnetic resonance imaging, and haemodynamic studies). The results demonstrate that three doses of MSCs improved both load-dependent and independent indices of LV function and reduced myocardial hypertrophy and fibrosis; in contrast, one dose failed to produce most of these benefits.

**Conclusions:**

To our knowledge, this is the first study to show that intravenous infusion of a cell product improves LV function and structure in a large animal model of chronic ischaemic cardiomyopathy and that repeated infusions are necessary to produce robust effects. This study, conducted in a clinically relevant model, supports a new therapeutic strategy based on repeated intravenous infusions of allogeneic MSCs and provides a foundation for a first-in-human trial testing this strategy in patients with chronic ischaemic cardiomyopathy.

**Time of primary review: 37 days**

**See the editorial comment for this article ‘Coming around again’, by C. Savko and M.A. Sussman, https://doi.org/10.1093/cvr/cvae219.**

## 1. Introduction

Despite therapeutic advances, the prognosis of heart failure (HF) secondary to chronic ischaemic cardiomyopathy remains poor.^1^ The prevalence of this deadly syndrome is high (>3 million Americans) and continues to rise, making it a major health problem and an unmet medical need.^1^ Several clinical trials suggest beneficial effects of transendocardial injection of mesenchymal stromal cells (MSCs) in patients with ischaemic cardiomyopathy^2–6^ (reviewed in Bolli *et al*.^7^). However, the transendocardial route of cell delivery used in these studies is logistically complex, expensive, contraindicated in many clinical settings, and carries significant risks.^7^ Importantly, it is not suitable for repeated administrations, effectively limiting therapy to one dose.

Intravenous delivery of cells would overcome these limitations.^8^ In a rat model of chronic ischaemic cardiomyopathy, we have previously shown that intravenous infusions of MSCs improve LV function,^9^ despite the fact that few MSCs reach the heart via this route. This implies that the amelioration of cardiac function was due to actions of MSCs lodged in extracardiac organs (e.g. lung, spleen, and other tissues^8,10^). The well-known systemic anti-inflammatory actions of these cells may be particularly important, because persistent systemic inflammation appears to be associated with, and play a central role in, the progression of HF.^11–14^ MSCs possess immunomodulatory and anti-inflammatory properties, including suppression of native and adaptive immunity.^8,15–17^ These actions have been demonstrated not only in animal and human studies of HF,^18^ but also in many non-cardiac conditions.^17,19^ Of interest, intravenously delivered MSCs trapped in extracardiac tissues have been shown to exert systemic anti-inflammatory actions mediated by the release of immunomodulatory signals, leading to reduced myocardial inflammation^20–22^ (reviewed in Wysoczynski *et al*.^8^). However, aside from our rat study,^9^ the impact of intravenous administration of MSCs (or any cell type) on chronic ischaemic cardiomyopathy has not been examined in large animal models.

Furthermore, although a single intravenous infusion of MSCs may produce some benefit, multiple doses are more likely to be effective. Since cells (regardless of cell type) disappear shortly after transplantation,^10,23^ it seems self-evident that their actions will be limited in time, so that administering one dose of cells cannot be considered an adequate test of their efficacy.^7,8,24^ Thus, we reasoned that for the full therapeutic effects of intravenous MSC delivery to become apparent, it is necessary to use repeated doses in order to replace the cells that disappear. Although previous studies in rodent and pig models of HF have shown that repeated intramyocardial or intracoronary injections of cells are more effective than a single injection,^25–31^ (reviewed in Wysoczynski *et al*.^8^ and Bolli^24^), repeated intravenous administrations have not been tested thus far.

Based on these considerations, we hypothesized that (i) intravenous delivery of MSCs may be a useful strategy in ischaemic cardiomyopathy and (ii) the beneficial actions of MSCs are significantly augmented by repeated administrations. The objective of this study was to test these concepts in a rigorous manner in a preclinical, translationally relevant model of ischaemic cardiomyopathy. To this end, we examined the effects of either one or three intravenous infusions of MSCs in pigs with chronic ischaemic cardiomyopathy induced by a large myocardial infarction (MI). The results show that intravenous delivery of MSCs improves LV function and structure, and that three infusions produce greater improvement than a single infusion.

## 2. Methods

### 2.1 Induction of MI

All animal experiments were performed in accordance with *the Guide for the Care and Use of Laboratory Animals* published by the US National Institutes of Health (Eighth Edition, Revised 2010) and with the guidelines of the Animal Care and Use Committee of the University of Louisville, School of Medicine (Louisville, KY). Thirty-nine female Göttingen minipigs (average age, 11-month-old; 21.0 ± 0.8 kg) were purchased from Marshall BioResources (North Rose, NY). Pigs received orally 200 mg amiodarone daily for 7 days before MI as antiarrhythmic prophylaxis (see Supplementary material online, *Figure S1*). On the day of MI, pigs were premedicated with im ketamine (20 mg/kg) and xylazine (2 mg/kg). After induction of anaesthesia with iv propofol (1.5 mg/kg), animals were intubated and mechanically ventilated, and anaesthesia was maintained with 1–3% isoflurane supplemented with a mixture of 50% O_2_/50% N_2_. The right carotid artery was cannulated with an 8 French arterial sheath and a 7 French Hockey-stick catheter was advanced to the left main coronary artery ostium under fluoroscopy. A 3.0–3.5 × 20 mm PTCA balloon catheter (Boston Scientific, Natick, MA) was positioned in the proximal LAD, above the first diagonal branch (see Supplementary material online, *Video A*), so that the entire LAD territory would be rendered ischaemic. Balloon position was verified by contrast dye injection (Isovue-370) and documented by cineangiography prior to inflation. MI was induced by inflating the balloon for 150 min.^32^ Complete cessation of flow in the LAD was confirmed by angiography (see Supplementary material online, *Video A*). Amiodarone (4 mg/kg bolus over 20 min followed by 1.2 mg/kg/h infusion) and lidocaine (2 mg/kg bolus followed by 2 mg/kg/h infusion) were infused iv throughout the coronary occlusion. Cardioversion was performed by ‘hands free’ defibrillation at 300–360 J (HP Codemaster XL+ defibrillator). After 150 min, the balloon was deflated to allow reperfusion. The carotid artery was repaired by suture, and the neck incision was closed in layers. Three additional female Göttingen minipigs served as naïve pigs.

### 2.2 Bone marrow stromal cell isolation and culture

Bone marrow (BM) was harvested under aseptic conditions from the femur bones of a male Göttingen minipig. BM cell suspensions were seeded on T225 flasks using MEM medium (Gibco) supplemented with 20% FBS (Seradigm), 0.2 mM L-glutamine (Gibco), and 100 U/mL penicillin/streptomycin (Gibco). After 24 h, floating cells were removed and adherent cells were washed with PBS and suspended in growth medium for further *in vitro* expansion. At 70% confluence, BM MSCs were harvested and cryopreserved. BM MSCs were not used beyond passage 6. BM MSCs were maintained under standard incubation conditions at 37°C with 5% atmospheric CO_2_ and passaged using 0.25% trypsin-EDTA (ThermoFisher Scientific) when approaching ∼70% confluence.

### 2.3 Proliferation and cell population doubling time

Cell proliferation was assessed by counting cells with a BD LSRFortessa flow cytometer. Briefly, 20 000 cells were seeded per well in complete growth medium in 12-well plates. Twenty-four hours later, a group of cells was counted and recorded as time = 0 h. The medium in the remaining plates was then replaced with fresh growth medium, and the cells were cultured for the indicated durations. The cells were harvested by trypsinization and suspended in growth medium. A small volume of suspended cells was used to record cell events with the BD LSRFortessa flow cytometer, with proper gating to exclude cell debris. To calculate cell doubling time (Td), the following formula was used: Td = Tln2/ln(Xe/Xb), where T is the incubation time, Xb is the cell number at the beginning of the incubation time (i.e. t = 0 h), and Xe is the cell number at the end of the incubation time; Xe and Xb were recorded in the log phase of cell growth.

### 2.4 Flow cytometry

Cells were detached from culture dishes with 0.25% trypsin-EDTA. After incubation for 30 min at 4°C with monoclonal antibodies (see Supplementary material online, *Table S1*), cells were washed, suspended in 0.5 mL PBS, and analysed by flow cytometry with the BD LSRFortessa system. Post-acquisition analysis to determine expression of detected markers was performed with FlowJo software.

### 2.5 Treatment protocol

Thirty days after MI, pigs were randomly assigned to three groups that received, at 5-week intervals: (1) three doses of vehicle (vehicle group); (2) one dose of MSCs and two doses of vehicle (single-dose group); or (3) three doses of MSCs (repeated-dose group). MSCs were suspended in PBS (2 million cells/mL) for administration. Vehicle (PBS) and MSCs (2 million cells/kg) were infused intravenously at 1 mL/kg over a 30 min period. Pigs were euthanized 5 weeks after the 3rd treatment (see Supplementary material online, *Figure S1*). Euthanasia was performed in accordance with the guidelines of the Animal Care and Use Committee of the University of Louisville and was conducted after the final haemodynamic study. Pigs were heparinized (100 U/kg, iv), and deeply anaesthetized with 5% isoflurane. A bolus of 3–6 mL/kg of 3 M KCl solution was injected into the LV cavity via the pigtail catheter until the heart was arrested. Asystole was confirmed from the ECG. After cessation of electrical activity, the heart was harvested for post-mortem perfusion.

### 2.6 Echocardiographic studies

Pigs underwent five echocardiographic studies at the following time points: baseline (prior to induction of MI), pre-treatment (pre-Rx, 30 days after MI, immediately before the 1st treatment), and 35 days after the first (post-1st Rx), second (post-2nd Rx), and third (post-3rd Rx) treatments (see Supplementary material online, *Figure S1*). Pigs were sedated and lightly anaesthetized with isoflurane using a mask. Body temperature was carefully monitored with a rectal probe and maintained within the physiologic range with a heating blanket. Using a portable Chison SonoBook 9 ultrasound machine, 2D parasternal LV long-axis images were acquired at the right parasternal location^33,34^ with a P2-V phase array probe (see Supplementary material online, *Video B*). End-diastolic and systolic volumes (EDVs and ESVs) and LV ejection fraction (EF) were measured online using the onboard Chison SonoBook 9 Software and confirmed off-line using the NIH ImageJ software.

### 2.7 Cardiac magnetic resonance imaging (MRI)

MRI was performed at pre-treatment (pre-Rx, 30 days after MI, immediately before the 1st treatment) and at the end of the study (post-Rx, 35 days after the 3rd treatment, immediately before euthanasia) (see Supplementary material online, *Figure S1*) using a 3.0 T clinical scanner (Magnetom, Siemens AG, Munich, Germany).^32,35–37^ EDV, ESV, stroke volume (SV), EF, longitudinal segmental strain, radial strain, longitudinal global strain, radial global strain, and global strain rate were measured as described.^38,39^

### 2.8 Haemodynamic studies

Haemodynamic studies were performed at pre-treatment (pre-Rx, 30 days after MI, immediately before the 1st treatment) and at the end of the study (post-Rx, 35 days after the 3rd treatment, immediately before euthanasia) (see Supplementary material online, *Figure S1*). Briefly, under isoflurane anaesthesia, a 7 F sheath was introduced into the right carotid artery and a 9 F sheath into the right external jugular vein. Under fluoroscopic guidance, an 8 F venous balloon (PTS sizing balloon, 20 mm × 30 mm) was placed in the inferior vena cava (IVC) and a 5 F Scisense variable segment length (VSL) pressure–volume (PV) catheter with an ADV500 PV system (Transonic) was advanced into the LV cavity to record LV pressure and admittance-derived true-volume signals.^40^ To assess contractility, PV loops were recorded during steady state and during brief IVC occlusions. The LabChart Pro v8.0.10 software (AD Instruments Inc.) was used for data analysis.

### 2.9 Gross pathology

After euthanasia, the heart was harvested, weighted, and sliced transversely into 6–7 sections that were incubated in 1% triphenyl tetrazolium chloride (TTC) at 37°C for 5 min. After TTC staining, the right ventricle was removed and LV sections were weighted and photographed. Scar size was measured by planimetry.^41^ Tissue samples were obtained from the risk, border, and non-infarcted regions for pathologic analysis.

### 2.10 Histologic studies

The protocols for histologic analyses have been described.^9,25,27,42–44^ Briefly, formaldehyde-fixed, paraffin-embedded myocardial samples were sectioned in 5 µm slices and mounted on positively charged glass slides. Histologic and immunofluorescent stainings were performed following manufacturer's protocols.

All images were captured using a Nikon Eclipse 80i fluorescence microscope (Nikon, Tokyo, Japan) with a ×20 objective. Representative images were taken with the same ×20 objective, unless otherwise specified. Digital imaging and quantifications were performed using NIS Elements Software (Nikon, Tokyo, Japan). Histological and fluorescent stains and antibodies were used to identify specific cell markers and compartments. Haematoxylin and eosin staining was used to determine proper tissue orientation for subsequent measurements. Myocardial collagen content was quantitated using picrosirius red staining. Cell death was enumerated by TUNEL staining using the In Situ Cell Death Detection kit (Millipore Sigma, Rockville, MD). Fluorescein-labelled Isolectin B4 (Griffonia simplicifolia Lectin I, Vector Labs, Newark, CA) staining was performed to determine capillary density. Rhodamine-conjugated wheat germ agglutinin (WGA, Vector Labs, Newark, CA) staining was performed to outline cell membranes and facilitate measurements of cardiomyocyte cross-sectional area. The total number of myocytes measured in the three groups was 22 784 in the risk zone, 32 914 in the border zone, and 36 896 in the non-ischaemic zone. Haematopoietic cells and macrophages were identified by antibodies against CD45 (ab10558) and CD68 (ab201340), respectively, and counterstained with species-specific fluorescent secondary antibodies. Nuclei were stained with DAPI (4′,6-diamidino-2-phenylindole).

### 2.11 Statistical analysis

All data are expressed as means ± S.e.m. LV functional data were analysed with two-way repeated-measures analysis of variance (ANOVA), where treatment group and time points were considered as fixed effects, and subjects were considered random effects. *Post hoc* Student's *t*-tests with Bonferroni correction were used to evaluate treatment effects at the same time point, as well as to evaluate time effect within the same treatment group. Cardiomyocyte size was analysed with Kruskal–Wallis one-way ANOVA on ranks followed by pairwise multiple comparisons with Dunn's method. Morphometric, histologic, immunohistochemical, and haemodynamic data were analysed by one-way ANOVA followed by Student's *t*-tests with Bonferroni correction for inter-group comparisons.^9,25,27,42,43^ All analyses were conducted with SigmaStat 3.5. A *P* value of <0.05 was considered significant.

## 3. Results

A total of 39 Gottingen minipigs were enrolled and assigned to three groups (*n* = 13/each). Nine pigs were excluded from the final data analysis (see Supplementary material online, *Table S2*): four died of ventricular fibrillation (two in the vehicle and one each in the single-dose and repeated-dose groups); four were euthanized (one in the vehicle group due to severe breathing problems, two in the single-dose, and one in the repeated-dose groups due to gastrointestinal issues); and one pig in the repeated-dose group was excluded because of failure of the balloon occluder. Thus, a total of 30 pigs were included in the final analysis (10 in each of the three groups) (see Supplementary material online, *Table S2*).

**Figure 1:**
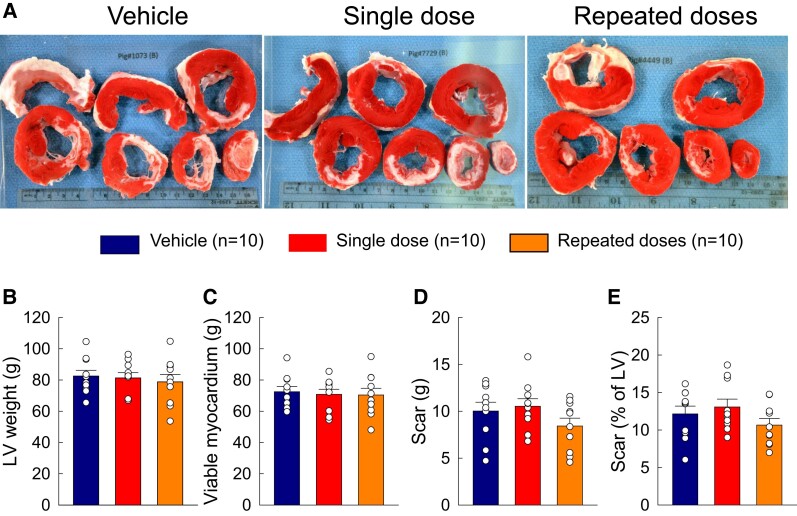
Gross pathology. (*A*) Representative LV sections from vehicle-, single-dose-, and repeated-dose-treated pigs stained with TTC, showing scar (white) and viable myocardium (red). (*B*–*D*) Post-mortem analysis of LV mass (*B*), viable myocardium mass (*C*), scar (g) (*D*), and scar as a percentage of LV mass (*E*). Open circles represent individual pigs and bars/whiskers mean ± S.e.m.

Age, body weight, number of defibrillation shocks, and days between treatments were similar among the groups (see Supplementary material online, *Table S3* and Supplementary material online, *Figure S3*). At post-mortem examination, there were no significant differences with respect to spleen and total heart weight (data not shown). The weight of the left ventricle, LV viable myocardium, and LV scar did not differ among groups (see Supplementary material online, *Table S3* and *Figure 1*).

### 3.1 Characterization of MSCs

For the experiments described herein, cells exhibiting plastic adherence to tissue-culture grade dishes were used following their propagation in culture for no more than five passages. BM MSCs displayed a mesenchymal immunophenotype characterized by positivity for CD90, CD29, CD105, and CD44, but absence of endothelial specific CD31 and haematopoietic CD45 markers (see Supplementary material online, *Figure S2A*). When propagated in tissue-culture grade dishes, MSCs showed typical BM MSCs linear cell growth, with a population doubling time of 37.5 h (see Supplementary material online, *Figure S2B*).

### 3.2 Effect of MSCs on LV function measured by echocardiography

**Figure 2:**
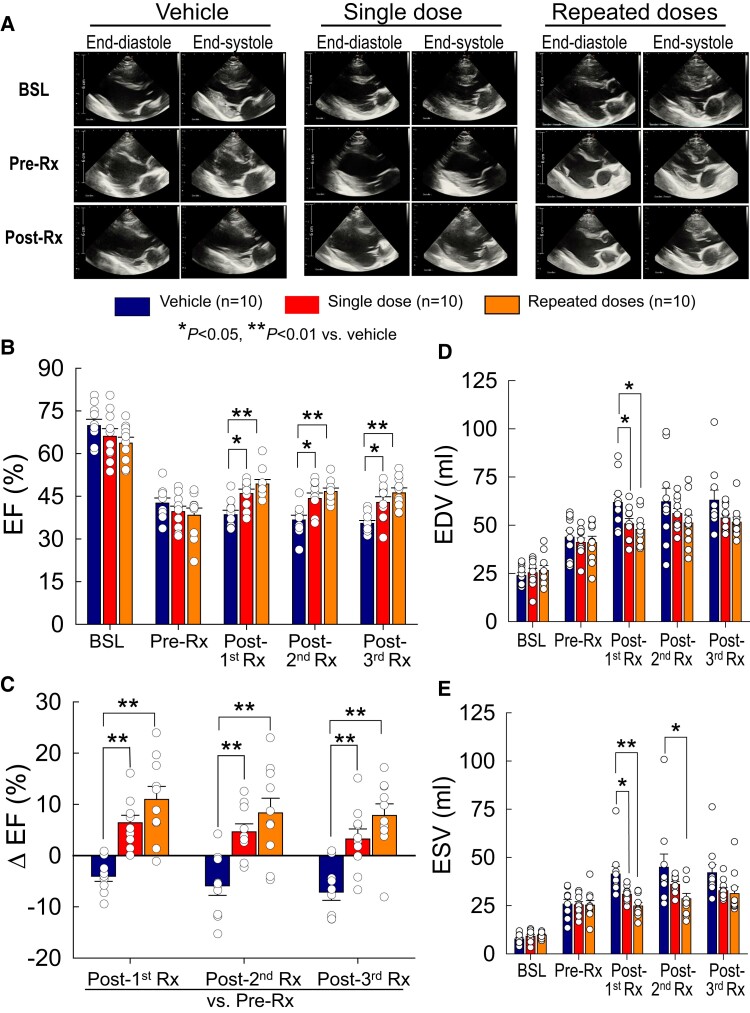
LV function assessed by echocardiography. (*A*) Representative 2D long-axis views at end-systole and end-diastole from vehicle-, single-dose-, and repeated-dose-treated pigs at baseline (BSL), 30 days after MI (immediately before the 1st treatment, pre-Rx), and at 105 days after the 1st treatment (35 days after the 3rd treatment, immediately before euthanasia, post-Rx). (*B*) LV EF at baseline (BSL), 30 days after MI (immediately before the 1st treatment), 35 days after the 1st treatment (post-1st Rx), 35 days after the 2nd treatment (post-2nd Rx), and 35 days after the 3rd treatment (post-3rd Rx) (immediately before euthanasia). (*C*) Δ EF at 35 days after the 1st, 2nd, and 3rd treatments vs. pre-treatment (pre-Rx). (*D* and *E*) LV end-diastolic volume (EDV) and end-systolic volume (ESV) at the same time points as in panel *B*. Open circles represent individual pigs and bars/whiskers group mean ± S.e.m. *P < 0.05, **P < 0.01 (two-way repeated-measures ANOVA followed by Bonferroni multiple comparison tests, *n* = 10).

Echocardiographic measurements are summarized in Supplementary material online, *Table S4*; representative echocardiographic images and videos are provided in *Figure 2A* and Supplementary material online, *Video B*. As shown in *Figure 2*, at baseline LV EF, EDV, ESV, and SV were similar among groups. At 30 days after MI (before treatment, pre-Rx), EF decreased >25 units from baseline values (before MI) in all pigs; the average decrease was similar among groups (−27.2 ± 2.1, −26.4 ± 1.8, and −25.3 ± 2.7 EF units, respectively, in the vehicle, single-dose, and repeated-dose groups) (see Supplementary material online, *Table S4*). As expected, after MI EDV and ESV increased in all groups and, again, the average changes were similar (*Figure 2* and Supplementary material online, *Table S4*). These data indicate that both the loss of contractile function caused by MI and the severity of post-MI LV dysfunction before start of treatment were comparable among the three groups.

At 35 days after the first treatment (post-1st Rx), the vehicle-treated group showed further deterioration of LV function and dimensions (*Figure 2*), as documented by a decline in EF and an increase in EDV and ESV compared with pre-treatment (pre-Rx) values at 30 days after MI (*Figure 2* and Supplementary material online, *Table S4*). In contrast, during the same interval, LV function improved in the treated groups, so that, at 35 days after the first treatment, EF was higher both in the single-dose (46.0 ± 1.5%, *P* < 0.05) and repeated-dose (49.3 ± 1.6%, *P* < 0.01) groups compared to the vehicle group (38.5 ± 1.6%) (*Figure 2B*). The change in EF vs. pre-treatment (Δ EF at post-1st Rx vs. pre-Rx) was also significantly greater both in the single-dose (+6.4 ± 1.5%, *P* < 0.01) and repeated-dose (+10.9 ± 2.5%, *P* < 0.01) groups vs., vehicle (−4.1 ± 1.0%) (*Figure 2C*). EDV was significantly reduced vs. vehicle both in the single-dose (50.7 ± 2.7 mL vs. 61.8 ± 4.2 mL, *P* < 0.05) and repeated-dose groups (47.8 ± 2.7 mL vs. 61.8 ± 4.2 mL, *P* < 0.05) (*Figure 2D*). Similarly, ESV was significantly reduced both in the single-dose (30.9 ± 1.2 mL, *P* < 0.05) and repeated-dose groups (24.8 ± 1.9 mL, *P* < 0.01) compared with vehicle (41.4 ± 3.9 mL) (*Figure 2E*).

After the second treatment, EF remained significantly higher both in the single-dose (44.2 ± 1.8%) and in the repeated-dose (46.6 ± 1.2%) groups compared with vehicle (36.6 ± 1.7%) (*P* < 0.05 and <0.01, respectively (*Figure 2B*). Likewise, Δ EF at post-2nd Rx vs. pre-Rx was significantly greater both in the single-dose (+4.6 ± 1.5%) and in the repeated-dose (+8.3 ± 2.9%) groups than in the vehicle group (−5.9 ± 1.9%, *P* < 0.01 for both comparisons) (*Figure 2C*). Compared with vehicle (44.9 ± 6.8 mL), ESV was reduced in the repeated-dose group (28.5 ± 2.8 mL, *P* < 0.01) but not in the single-dose group (36.0 ± 1.5 mL) (*Figure 2E*).

After the third treatment (at the end of the study), EF was still significantly higher both in the single-dose (42.8 ± 2.0%) and in the repeated-dose (46.2 ± 1.7%) groups vs. vehicle (35.4 ± 1.0%, *P* < 0.05 and <0.01, respectively) (*Figure 2B*), even though pigs in the single-dose group received MSCs only at the 1st treatment. Although EF was numerically greater (∼4 EF units) in the multiple-dose than in the single-dose group, the difference between the two treated groups was not statistically significant. Δ EF at post-3rd Rx vs. pre-Rx was also greater both in the single-dose (+3.2 ± 2.0%) and in the repeated-dose (+7.8 ± 2.3%) groups compared with vehicle (−7.1 ± 1.6%, *P* < 0.01 for both) (*Figure 2C*). EDV and ESV did not differ significantly among the three groups, although they tended to be lower in the two cell-treated groups (*Figure 2D* and *E*). However, the Δ ESV [change in ESV within a group vs. pre-treatment (pre-Rx)] was statistically different between vehicle and single-dose groups and between vehicle and repeated-dose groups after both the first and third treatments; after the second treatment, only the repeated-dose group was significantly different from vehicle (see Supplementary material online, *Table S4*).

In summary, the echocardiographic data indicate that a single intravenous dose of MSCs improved LV function compared with vehicle at 105 days after treatment and that three doses produced a numerically, but not statistically, greater improvement.

### 3.3 Effect of MSCs on LV function measured by MRI

**Figure 3:**
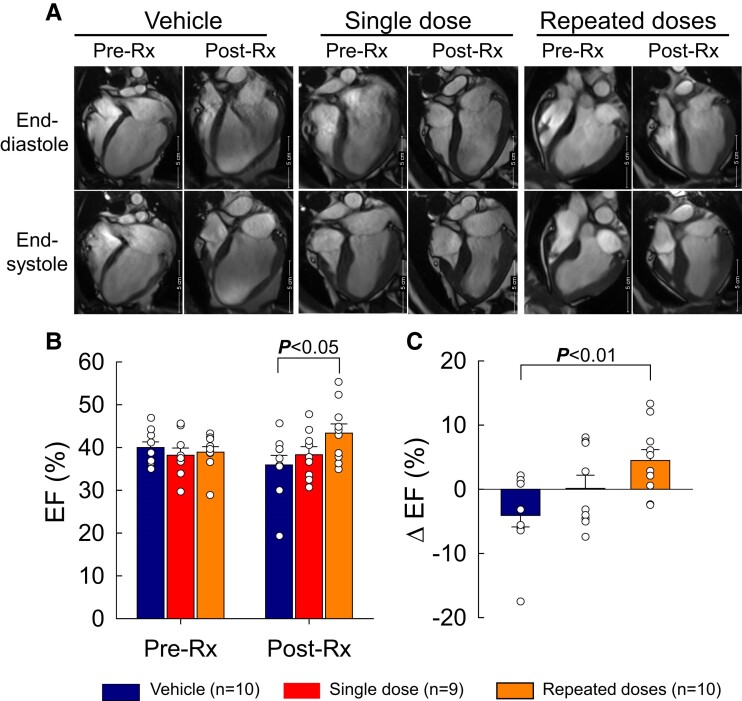
LV function (EF) assessed by MRI. (*A*) Representative four-chamber views at end-systole and end-diastole from vehicle-, single-dose-, and repeated-dose-treated pigs at 30 days after MI (before the 1st treatment, pre-Rx) and at 105 days after the 1st treatment (immediately before euthanasia, post-Rx). (*B*) LV EF at pre-Rx and post-Rx. (*C*) Δ EF at post-Rx vs. pre-Rx. Open circles represent individual pigs and bars/whiskers mean ± S.e.m. *P* values shown for absolute EF values (two-way repeated-measures ANOVA followed by Bonferroni multiple comparison tests, *n* = 9/10) and for Δ EF (one-way ANOVA followed by Bonferroni *t*-tests, *n* = 9/10).

All pigs underwent two MRI scans (pre-Rx and post-Rx), except for one pig in the single-dose group that could not be imaged because of COVID-19-related shut down of the MRI facility. The MRI data are summarized in Supplementary material online, *Table S5*, and representative videos are shown in Supplementary material online, *Videos C* and *D*. As shown in *Figure 3* and Supplementary material online, *Table S5*, EF, EDV, ESV, and SV were similar among groups before treatment (pre-Rx). After the 3rd treatment [post-Rx (end of study)], EF decreased in the vehicle group to 35.9 ± 2.3% from 40.0 ± 1.3% at pre-Rx (*P* < 0.05), and was preserved in the single-dose group (38.3 ± 1.9% vs. 38.2 ± 1.7% at pre-Rx); in contrast, it increased significantly in the repeated-dose group (43.5 ± 2.1% vs. 38.9 ± 1.3% at pre-Rx, *P* < 0.05) (*Figure 3B*). As a result, at the end of the study, EF was significantly higher in the repeated-dose compared with the vehicle group (*Figure 3B*, *P* < 0.05); Δ EF was also significantly higher (+4.6 ± 1.7% vs. −3.4 ± 2.0%, respectively, *P* < 0.01) (*Figure 3C*). LV volumes, including EDV, ESV, and SV, increased from pre-Rx values in all groups, with no significant differences among them (see Supplementary material online, *Table S5*).

**Figure 4:**
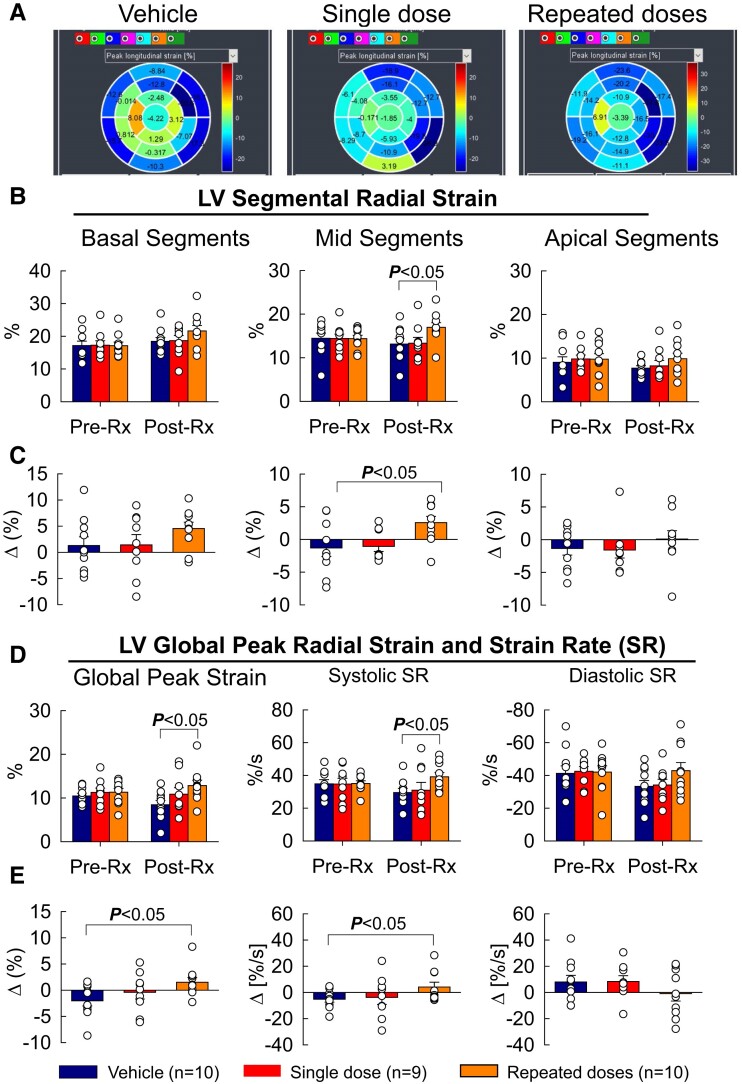
LV function (strain) assessed by MRI. (*A*) Representative bullseyes of LV 17-segment peak longitudinal strain from vehicle-, single-dose-, and repeated-dose-treated pigs at post-Rx (immediately before euthanasia). (*B*) Radial strain of basal, mid, and apical LV segments at 30 days after MI (before the 1st treatment, pre-Rx) and at 105 days after the 1st treatment (immediately before euthanasia, post-Rx). (*C*) Δ radial strain at post-Rx vs., pre-Rx in basal, mid, and apical segments. (*D*) LV global peak radial strain (left), global systolic strain rate (middle), and global diastolic strain rate (right). (*E*) Δ LV global peak radial strain (left), Δ systolic radial strain rate (middle), and Δ diastolic radial strain rate (right) at post-Rx vs. pre-Rx. Open circles represent individual pigs and bars/whiskers mean ± S.e.m. *P* values shown for absolute strain values (two-way repeated-measures ANOVA followed by Bonferroni multiple comparison tests, *n* = 9/10) and for Δ values (one-way ANOVA followed by Bonferroni *t*-tests, *n* = 9/10).

Myocardial strain analysis was performed by tracking the LV wall borders of long axis two-, three-, and four-chamber cine images using the Segment Medviso software (MedViso Segment CMR v3.3 R9405d, Lund, Sweden) (see Supplementary material online, *Video E*). LV segmental longitudinal (LS) and radial (RS) strain, LV global peak longitudinal and radial strain and strain rate, and the Δ segmental strain, global peak strain, and peak strain rate at post-Rx vs. pre-Rx are summarized in *Figure 4*, Supplementary material online, *Table S6A* and *B*, and Supplementary material online, *Figure S4*. Before treatment (pre-Rx), LS and RS in the basal, mid, and apical segments as well as LV global peak strain were comparable among groups. MSC administration had no discernible effect on LS (Supplementary material online, *Figure S4*). However, repeated intravenous injections of MSCs improved segmental RS numerically in the basal and apical segments, and the improvement was significant (*P* < 0.05) in the mid segments (*Figure 4B* and *C*). Similarly, repeated intravenous injections significantly improved LV global peak RS and systolic peak strain rate (*Figure 4D* and *E*); in contrast, a single MSC injection had no effect on segmental RS and LV global peak RS and strain rate (*Figure 4*).

### 3.4 Effect of MSCs on LV function measured by haemodynamic studies

Haemodynamic parameters are summarized in Supplementary material online, *Table S7*. As was the case with echocardiographic and MRI measurements, before treatment (pre-Rx), all haemodynamic variables [EDV, ESV, SV, EF, cardiac output (CO), end-systolic pressure, end-diastolic pressure, dP/dt_max_, dP/dt_min_, Tau, end-systolic elastance (Ees), and end-diastolic elastance (Eed)] were comparable among groups. At the end of the study (post-Rx), dP/dt_max_ was significantly improved in the repeated-dose group (1164 ± 61 mmHg/s vs. 1019 ± 46 mmHg/s at pre-Rx, *P* < 0.05) but was not significantly different among groups. The increase in ESV, EDV, and Eed was numerically smaller in pigs receiving multiple MSC doses than in those receiving vehicle, but the differences were not statistically significant.

**Figure 5:**
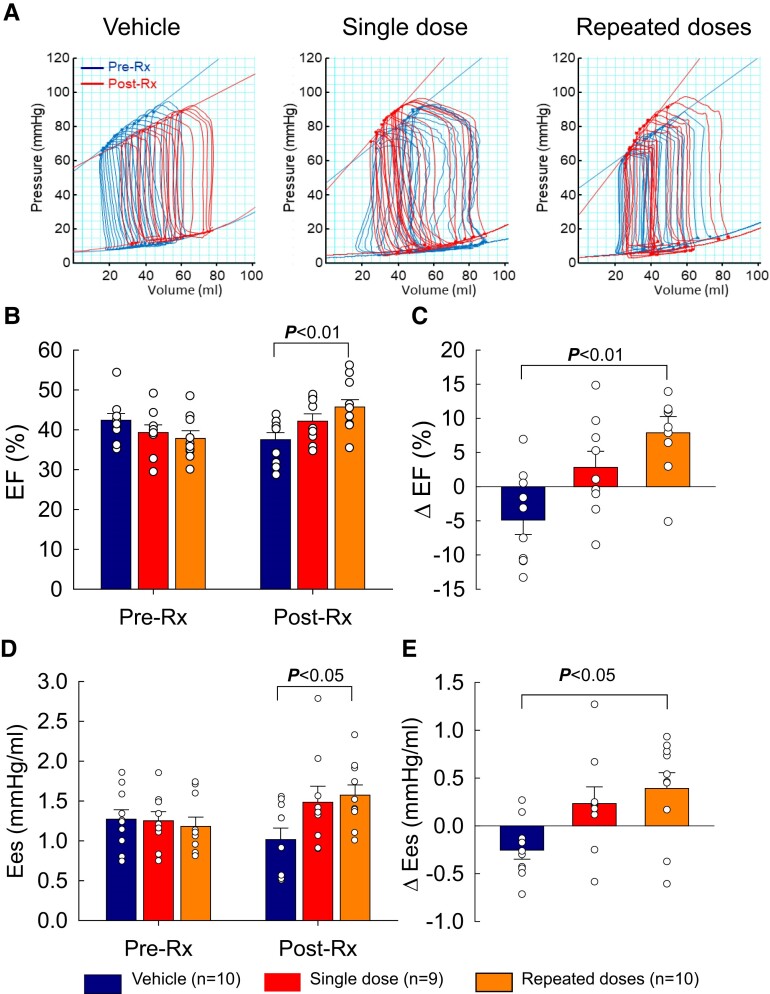
LV function assessed by haemodynamic studies. (*A*) Representative pressure–volume (PV) loops from vehicle-, single-dose-, and repeated-dose-treated pigs at 30 days after MI (before the 1st treatment, pre-Rx) and at 105 days after the 1st treatment (immediately before euthanasia, post-Rx). (*B*) LV EF at pre-Rx and post-Rx. (*C*) Δ EF at post-Rx vs. pre-Rx. (*D*) Elastance (EeS) at pre-Rx and post-Rx. (*E*) Δ elastance at post-Rx vs. pre-Rx. Open circles represent individual pigs and bars/whiskers mean ± S.e.m. *P* values shown for absolute values (two-way repeated-measures ANOVA followed by Bonferroni multiple comparison tests, *n* = 9/10) and for Δ values (one-way ANOVA followed by Bonferroni *t*-tests, *n* = 9/10).

EF (a load-dependent index of LV function) decreased in the vehicle group to 37.5 ± 1.8% at post-Rx from 42.4 ± 1.7% at pre-Rx (Δ EF −4.9 ± 2.1%, *P* < 0.05), increased slightly in the single-dose group (42.1 ± 1.8% vs. 39.3 ± 1.9% at pre-Rx, *P* = NS), and increased significantly in the repeated-dose group (45.7 ± 2.1% vs. 37.8 ± 1.7% at pre-Rx, Δ EF +7.9 ± 2.4%, *P* < 0.01) (*Figure 5B* and Supplementary material online, *Table S7*). As a result, at the end of the study (post-Rx), both EF and Δ EF were greater in the repeated-dose than in the vehicle group (*P* < 0.01 for both; *Figure 5B* and *C*). Ees (a load-independent index of contractility) improved significantly from pre-Rx values in the repeated-dose group (1.57 ± 0.13 mmHg/mL at post-Rx vs. 1.18 ± 0.12 at pre-Rx, *P* < 0.05) but not in the single-dose group; in the vehicle group, Ees actually decreased (*Figure 5* and Supplementary material online, *Table S7*). The Δ Ees was greater than vehicle in the multiple-dose group (+0.39 ± 0.16 vs. −0.25 ± 0.09, respectively, *P* < 0.05) but not in the single-dose group. As a result, at the end of the study both Ees and Δ Ees were greater in the repeated-dose group than in the vehicle group (*P* < 0.05 for both, *Figure 5D* and *E*).

In summary, three independent methods (echocardiography, MRI, and haemodynamic studies) consistently indicated that repeated intravenous doses of MSCs improved LV function compared with vehicle treatment; in contrast, only one method (echocardiography) showed improved LV function after a single MSC dose.

### 3.5 Effect of MSCs on LV structure

**Figure 6:**
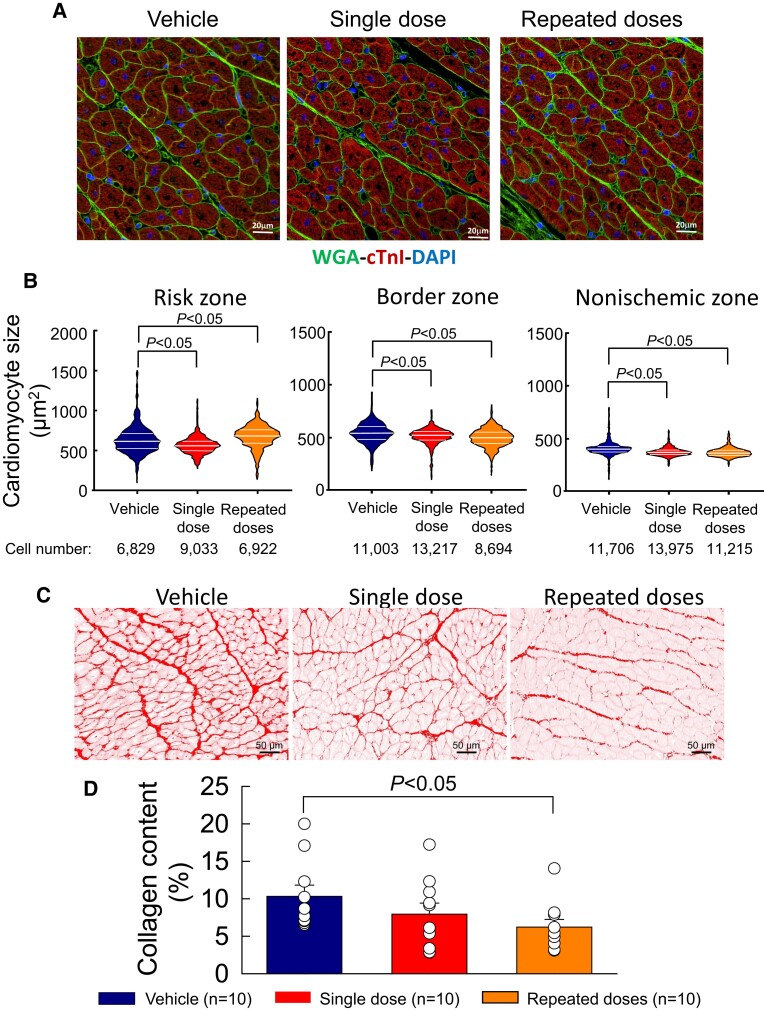
Cardiomyocyte size and myocardial collagen content. (*A*) Representative images of LV myocardium in the non-ischaemic region stained with WGA, cTnI, and DAPI from vehicle-, single-dose-, and repeated-dose-treated pigs. (*B*) Violin plot analysis comparing the distribution of cardiomyocyte cross-sectional area in the risk zone (left panel), border zone (middle panel), and non-ischaemic zone (right panel). Cell number is the total number of cells used for each plot from the 10 animals of each group. (*C*) Representative colour deconvoluted images of myocardium stained with Picrosirius red in the non-ischaemic LV region of vehicle-, single-dose-, and repeated-dose-treated pigs. (*D*) Collagen content measured as Picrosirius red positive area, expressed as a percentage of total measured area. Open circles represent individual pigs and bars/whiskers mean ± S.e.m. *P* values shown for cross-sectional area (Kruskal–Wallis one-way ANOVA on ranks followed by Dunn's all pairwise multiple comparison tests using cell numbers ranging from 6829 to 13 975 from *n* = 10) and for collagen content (one-way ANOVA followed by Bonferroni *t*-tests, *n* = 10).

Cardiomyocyte cross-sectional area was measured in LV sections stained with WGA-cTnl-DAPI in the risk, border, and non-ischaemic regions (*Figure 6A*). Cardiomyocyte cross-sectional area was greater in the risk region than in the non-ischaemic region in all groups (*Figure 6B*) In the risk region, cross-sectional area was smaller than vehicle in hearts receiving a single dose of cells and greater in those receiving multiple doses. However, in the border and non-ischaemic regions (which account for most of the contractile function of the left ventricle), cross-sectional area was consistently less in both the single- and multiple-dose groups compared with vehicle (*Figure 6B*). Furthermore, in the non-ischaemic region the variability in cross-sectional area was much less after one or three doses of cells than in control hearts (*Figure 6B*). These data indicate that intravenous administration of MSCs, even as a single dose, reduced the compensatory myocyte hypertrophy that occurs after MI.

Picrosirius red-stained LV sections (*Figure 6C*) were analysed to determine the effect of intravenous MSCs on myocardial collagen deposition. As shown in *Figure 6D*, collagen content in the non-ischaemic region was reduced by 23% after a single dose of MSCs, but the difference was not statistically significant (*P* = NS). However, three doses of MSCs significantly reduced collagen content in the non-ischaemic region by 40% vs. vehicle control (*P* < 0.05), indicating an antifibrotic action of therapy.

Apoptosis was analysed by TUNEL staining of LV sections (see Supplementary material online, *Figure S5A*). As shown in Supplementary material online, *Figure S5B*, the number of TUNEL positive cells in the non-ischaemic region tended to be lower in both the single-dose and the repeated-dose groups compared with the vehicle group, but the differences were of borderline statistical significance (*P* = 0.07 and 0.06, respectively).

**Figure 7:**
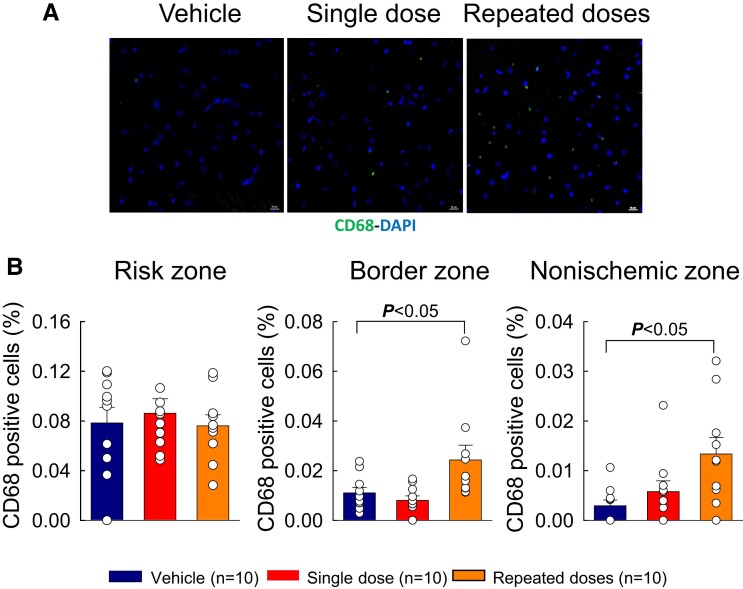
Myocardial content of CD68 positive cells. (*A*) Representative images of myocardium stained for CD68 in the non-ischaemic LV region of vehicle-, single-dose-, and repeated-dose-treated pigs. (*B*) Quantitative analysis of CD68 positive cells, expressed as a percentage of total DAPI positive cells, in the risk zone (left panel), border zone (middle panel), and non-ischaemic zone (right panel) of pigs that received vehicle (Vehicle group), a single MSC dose (Single dose group), or repeated MSC doses (Repeated doses group). Open circles represent individual pigs and bars/whiskers mean ± S.e.m. *P* values shown using one-way ANOVA followed by Bonferroni *t*-test, *n* = 10.

Immunostaining of LV sections was performed to analyse inflammatory cell infiltration. As shown in Supplementary material online, *Figure S6*, total CD45+ cell content did not differ among the three groups in the risk, border, or non-ischaemic zones. However, the number of CD68+ cells (macrophages) was significantly increased after three MSC doses compared with vehicle, both in the border zone (*P* < 0.05, *Figure 7C*) and in the non-ischaemic zone (*P* < 0.05, *Figure 7D*). There were no significant differences among groups with respect to capillary density (see Supplementary material online, *Table S8*).

## 4. Discussion

The goal of this translational study was to rigorously test the therapeutic efficacy of single or repeated intravenous infusions of MSCs in a clinically relevant porcine model of chronic ischaemic cardiomyopathy induced by a reperfused MI. A detailed and comprehensive analysis of LV function was performed with three independent modalities, i.e. echocardiography, cardiac MRI, and haemodynamic studies. The results can be summarized as follows: (i) when LV function was assessed by echocardiography, there was a significant improvement vs. placebo after a single dose of MSCs and a numerically, but not statistically, greater improvement after three doses; (ii) when LV function was assessed by MRI and haemodynamic studies, there was a significant improvement after three doses but not after a single dose; (iii) thus, only echocardiography showed improved LV function after one dose, whereas all three methods showed improvement after three doses; (iv) and three doses of MSCs, but not one, decreased myocardial fibrosis and hypertrophy and increased macrophage infiltration in the non-ischaemic region, without a reduction in scar size. Taken together, these data demonstrate that intravenous delivery of allogeneic MSCs has beneficial effects on the function and structure of the heart in a clinically relevant model of chronic ischaemic cardiomyopathy and that multiple doses are superior to a single dose. Previous studies in porcine models of HF have shown beneficial effects of a single transendocardial injection of MSCs^35,36^ or multiple transendocardial injections of skeletal myoblasts.^26^ However, to our knowledge, this is the first study to show that intravenous infusions of a cell product are beneficial and have cumulative effects in a large animal model of chronic ischaemic cardiomyopathy. The results support a new therapeutic strategy based on repeated intravenous infusions of MSCs and provide a robust rationale for testing this strategy in patients with ischaemic HF.

### 4.1 Methodological considerations

Studies in large animals, such as the pig, are expensive, time-consuming, and technically demanding; nevertheless, they are necessary to generate clinically relevant data that can be used as a foundation to proceed with clinical trials. Since the purpose of this work was to translate intravenous delivery of MSCs to patients, we elected to use a large animal (porcine) model of chronic ischaemic cardiomyopathy. We studied a reperfused MI, which is the type of MI commonly seen in clinical practice. The 40–50% drop in LV EF and Ees at pre-Rx (30 days after MI) vs. naïve pigs (see Supplementary material online, *Figure S7*) attests to the severity of LV dysfunction in this model. The dose of MSCs (2 million cells/kg) was selected because it is similar to the doses successfully used in many clinical trials of intravenous MSC administration in non-cardiovascular diseases^45,46^ (reviewed in Lanzoni *et al*.^47^). Given the translational nature of this study as a prelude to a clinical trial, it was not the purpose of this work to elucidate the molecular and cellular mechanisms that underlie the beneficial effects of MSCs. Large animals are not well-suited for mechanistic investigations for various reasons, including the scarcity of reagents, limited genetic manipulations, and the prohibitive cost.

This study was conducted according to current standards for rigour.^48,49^ Assignment to treatment groups was randomized and the investigators performing the experiments were blinded to group assignment until all functional data (echo, MRI, haemodynamics) had been collected and analysed. Only then was the treatment code broken and group assignment released to the investigators. These methods, and particularly the blinding of the investigators, are critical to avoid bias and ensure the validity of the results.^48,49^ The primary endpoint of this study was LV function. Consequently, an extensive, meticulous assessment of LV function was performed that included three independent methods (echocardiography, MRI, haemodynamic studies). Both load-dependent (e.g. EF) and independent (e.g. end-systolic elastance) parameters of cardiac function were examined. Although most studies have relied only on load-dependent variables such as EF, elastance is regarded as the gold standard for assessing myocardial contractility.^50^ Although echocardiography is relatively inexpensive and widely available, MRI offers superior accuracy in assessing LV volumes.^51^ Thus, this study used all three methods. Expecting identical results with three independent methods would be unrealistic and contrary to experience. Despite some inconsistencies, which are to be expected among different techniques, all three methods support the same two fundamental conclusions: (i) intravenous delivery of MSCs improves both load-dependent and independent indices of LV function, and (ii) three repeated doses are superior to one dose. The concordance among the results of three independent and different methods greatly strengthens our conclusions. Thus, we believe that this study offers a solid foundation for a first-in-human trial of intravenous MSC therapy in ischaemic cardiomyopathy.

### 4.2 Mechanism of action of MSCs

As mentioned above, the goal of this technically difficult, expensive, and time-consuming study was to promote translation of intravenous cell therapy to the clinical arena rather than to elucidate the mechanism of action of MSCs, which has proved elusive in previous studies and will require extensive work, considering the panoply of soluble factors (cytokines, chemokines, growth factors, bioactive lipids, non-coding RNAs, etc.) and exosomes released by MSCs in the surrounding tissue or blood stream.^7,8^ Since very few MSCs delivered by the intravenous route will reach the heart and in any case do not engraft,^8^ myocardial regeneration is not plausible. We found no evidence of scar reduction, as the mass of viable and scarred LV tissue did not differ between treated and control hearts (*Figure 1*). Of note, LV mass and scar mass were measured by direct post-mortem examination, which is the most accurate method. Our measurements of capillary density showed no differences among groups (see Supplementary material online, *Table S8*). We found no conclusive evidence of reduced apoptosis, although there was a trend towards reduction with MSCs (see Supplementary material online, *Figure S5*) and we examined only one time point (end of study). Thus, pro-angiogenic or anti-apoptotic actions of MSCs seem unlikely. However, we did observe a significant reduction in tissue fibrosis in the non-ischaemic zone of pigs that received repeated doses (but not in those that received one dose) (*Figure 6*), suggesting that the functional improvement in these animals may have been due, at least in part, to antifibrotic actions of MSCs.

The most plausible hypothesis is that the salubrious effects of systemic MSC administration were underlain by the systemic anti-inflammatory actions of these cells. Mounting evidence indicates that the progressive deterioration of LV function in chronic HF is caused, at least in part, by systemic inflammation resulting from activation of the immune system and causing infiltration of the myocardium by inflammatory cells (e.g. monocytes).^11–14^ The spleen in particular has been implicated as an important component of this response (cardiosplenic axis^11–14^). Accordingly, interventions that suppress systemic inflammation may alleviate myocardial inflammation and LV dysfunction.^8^ MSCs are known to exert robust immunomodulatory and anti-inflammatory actions,^8,15–17^ and intravenous delivery of these cells may target the spleen and other immune tissues more effectively than local cardiac delivery. Indeed, it has been shown that after intravenous infusion, MSCs trapped in the lungs, spleen, and other extracardiac tissues produce systemic anti-inflammatory effects leading to reduced myocardial inflammation (e.g. decreased myocardial infiltration by pro-inflammatory macrophages, decreased myocardial expression of pro-inflammatory cytokines such as IL-1β and IL-6, and increased expression of anti-inflammatory cytokines such as IL-10), either via paracrine actions on adjacent immune cells that are then released into the circulation, or via endocrine release of immunomodulatory and anti-inflammatory signals into the blood.^20–22,52,53^ Similarly, in the clinical arena, systemic administration of MSCs has been shown to have profound anti-inflammatory and immunosuppressive effects in numerous non-cardiovascular settings, including graft-vs.-host disease, multiple sclerosis, amyotrophic lateral sclerosis, systemic lupus erythematosus, chronic obstructive pulmonary disease, and Crohn's disease.^19^ Thus, considerable evidence supports the hypothesis that, in this study, intravenous infusion of MSCs improved LV function and structure by attenuating the systemic inflammation associated with chronic HF. Our measurements of CD68 positive cells (macrophages) actually show an increase in the non-ischaemic region of pigs treated with multiple MSC doses (*Figure 7*). We postulate that these macrophages may be a reparative phenotype that exerts antifibrotic and anti-inflammatory actions. Besides anti-inflammatory effects, it is conceivable that the release of the MSC secretome into the circulation may exert other beneficial actions (e.g. antifibrotic actions).^8^

### 4.3 Intravenous cell therapy

Conceptually, the use of the intravenous route for cell delivery is supported by two considerations. First, the rapid disappearance of cells after transplantation^10,23^ means that their functional benefits are not underlain by engraftment and formation of new cardiomyocytes, but instead by secretion of factors that act on the host myocardium in a paracrine or, in the case of intravenous delivery, endocrine fashion.^8–10^ Secondly, as detailed above, MSCs would be expected to be beneficial in HF because of their well-established systemic anti-inflammatory actions.^8^ Indeed, we have previously reported that a single intravenous administration of MSCs improved LV function in a rat model of chronic HF.^9^ Apart from chronic HF, intravenous delivery of MSCs has been found to impart beneficial effects in various animal models of acute MI, including mice,^20,54–56^ rats,^52,53,57–64^ rabbits,^65^ and swine.^66–71^ Major differences between this body of work and the present study include the fact that these previous studies^20,52–71^ examined acute MI, which is very different from chronic HF, and importantly, that all of them used a single cell dose.

### 4.4 Repeated cell doses

Thus far, almost all preclinical and virtually all clinical studies of cell therapy have used one dose of cells.^7,8,24^ Intuitively, expecting one dose of cells to be sufficient to bring about a therapeutic benefit seems unrealistic. It would appear more sensible to assume that just like pharmacologic therapies need to be repeated, so do cell-based therapies.^7,8,24^ These considerations motivated us to test the repeated-doses paradigm in a series of studies in rat^25,27^ and mouse^28–31^ models of chronic ischaemic cardiomyopathy in which cells were administered either once or thrice into the LV cavity (reviewed in Wysoczynski *et al*.^8^ and Bolli^24^). These investigations showed that three doses of cells had cumulative effects. i.e. the improvement in LV EF was greater than after a single dose.^25,27–31^ The present study is the next logical step towards translation. Compared with our previous work in rodents,^25,27–31^ the major advances are that repeated doses are superior to a single dose after intravenous delivery, which is the only feasible route for repeated treatments in patients; that this strategy is effective in a large animal model that mimics the clinical setting of chronic ischaemic cardiomyopathy; and that the benefits can be documented with MRI, the clinical gold standard to assess LV function.

Other investigations have also examined the utility of repeated doses in rodents,^72–74^ but these studies were conducted in models of acute MI, not chronic ischaemic cardiomyopathy, and none used the intravenous route for cell delivery. One study in a Gottingen minipig model of chronic MI^26^ reported that transendocardial injections of one, two, or three doses of autologous skeletal myoblasts at 6-week intervals resulted in a cumulative improvement in LV EF. The main differences between this and the present study are that we used bone marrow allogeneic MSCs and delivered them systemically rather than transendocardially. Skeletal myoblasts have now been abandoned in cardiovascular cell therapy.^7^

### 4.5 Clinical implications

The results of this study support two novel therapeutic strategies—intravenous cell delivery and repeated cell doses—both of which could be a significant advance in the treatment of patients with HF. Clinically, the use of repeated transendocardial cell injections is hindered by major safety, regulatory, financial, and logistic issues, not to mention patient adherence, making it extremely difficult, if not impossible, in clinical practice.^7^ In contrast, intravenous delivery of cells is exquisitely suitable for repeated cell administrations, even beyond three doses.

The use of the intravenous route would dramatically expand the HF population that could receive cell therapy. Compared with the invasive (transendocardial) route currently used in patients, intravenous delivery is simpler, less expensive, and much safer; thus, it would greatly broaden the utilization, feasibility, and affordability of cell therapy, making it possible to use it as an outpatient procedure in almost every medical centre and in almost every patient with HF while greatly reducing the complexity, cost, and risks of this treatment. Intravenous cell administration would also reduce disparities in health care for minorities who may have less access to expensive invasive treatments.

## 5. Conclusions

This study demonstrates that three doses of MSCs, given intravenously, improve both load-dependent and independent indices of LV function and reduce myocardial hypertrophy and fibrosis; in contrast, one dose failed to produce most of these benefits. To our knowledge, this is the first study to show that intravenous infusion of a cell product improves LV function and structure in a large animal model of chronic ischaemic cardiomyopathy and that repeated infusions are necessary to produce robust effects. These results, obtained in a clinically relevant model, support a new therapeutic strategy based on repeated intravenous infusions of allogeneic MSCs and provide a foundation for a first-in-human trial testing this strategy in patients with chronic ischaemic cardiomyopathy. The recently started CATO trial (NCT06145035) is the direct clinical translation of this preclinical pig study to humans, underscoring the translational value of the present observations.

## Supplementary Material

cvae173_Supplementary_Data

## Data Availability

Data are available on reasonable request.
