# Supplementary material for: Intravenous infusions of mesenchymal stromal cells have cumulative beneficial effects in a porcine model of chronic ischaemic cardiomyopathy
Source: Cardiovasc Res. 2024 Aug 20;120(15):1939–52. doi: 10.1093/cvr/cvae173 (PMC11630033; doi:10.1093/cvr/cvae173)
Supplement: cvae173_Supplementary_Data [file cvae173_supplementary_data.zip › Supplementary figures&Video legends- (07-08-24).pptx]

## Slide 1
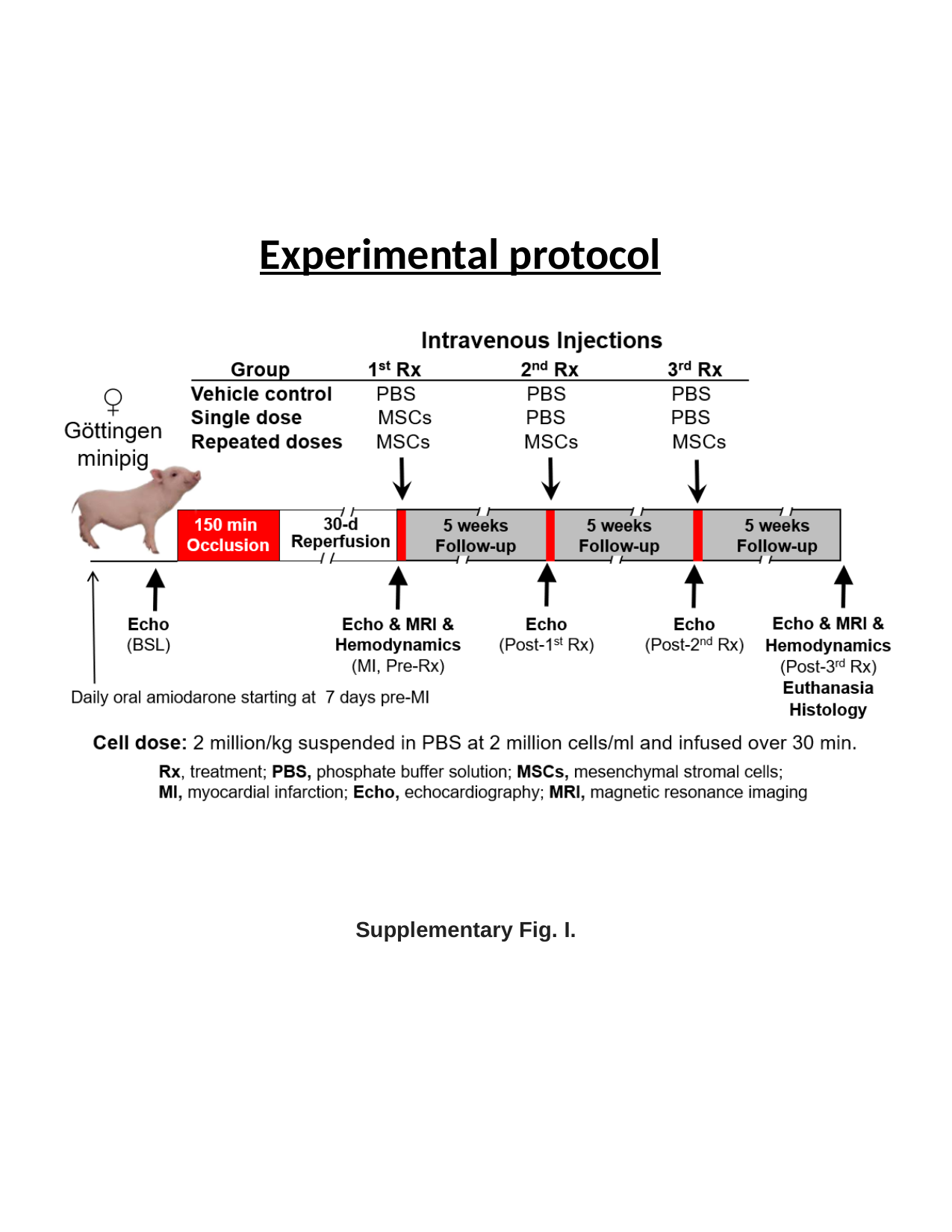

Experimental protocol
Supplementary Fig. I.

## Slide 2
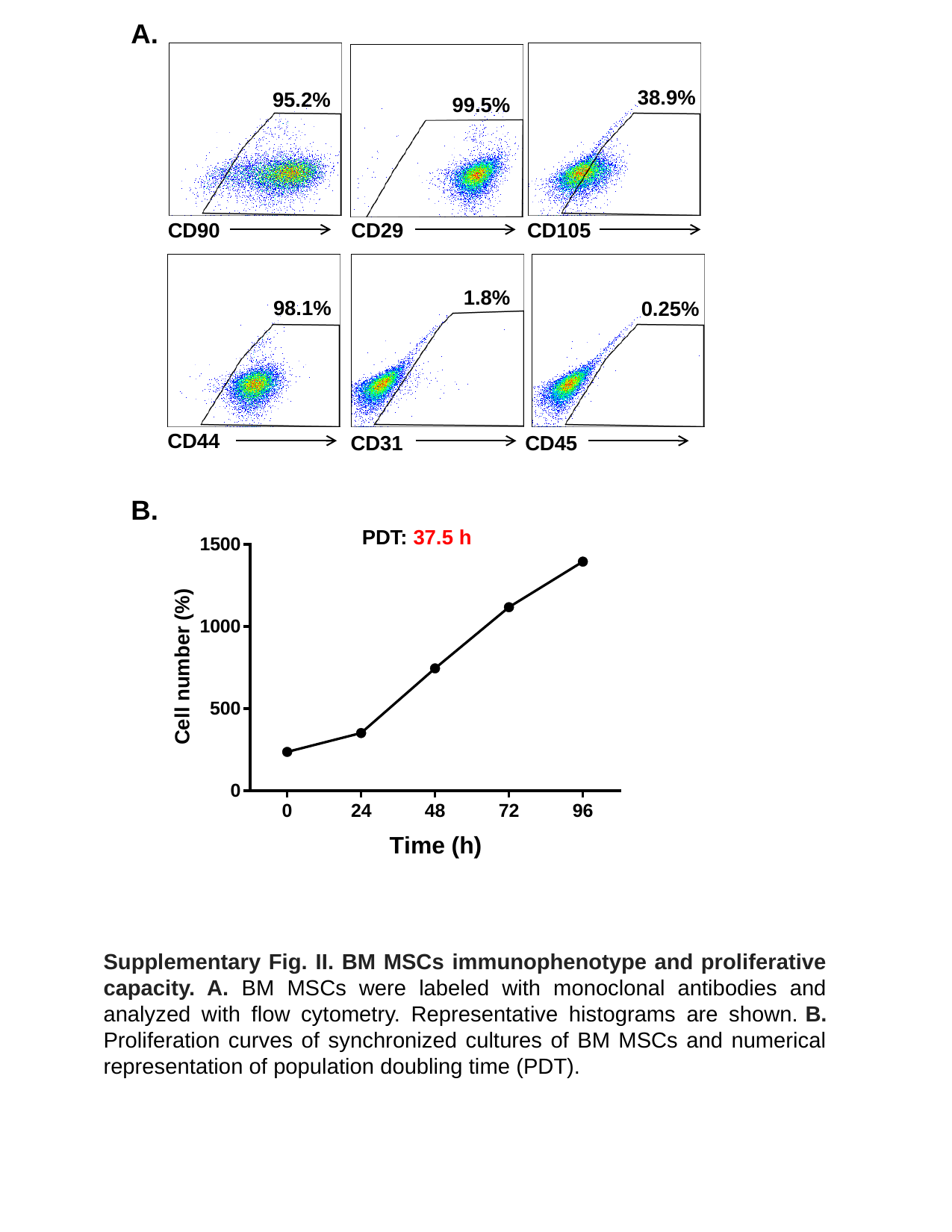

A.
38.9%
95.2%
99.5%
CD90
CD29
CD105
1.8%
98.1%
0.25%
CD44
CD45
CD31
B.
PDT: 37.5 h
Supplementary Fig. II. BM MSCs immunophenotype and proliferative capacity. A. BM MSCs were labeled with monoclonal antibodies and analyzed with flow cytometry. Representative histograms are shown. B. Proliferation curves of synchronized cultures of BM MSCs and numerical representation of population doubling time (PDT).

## Slide 3
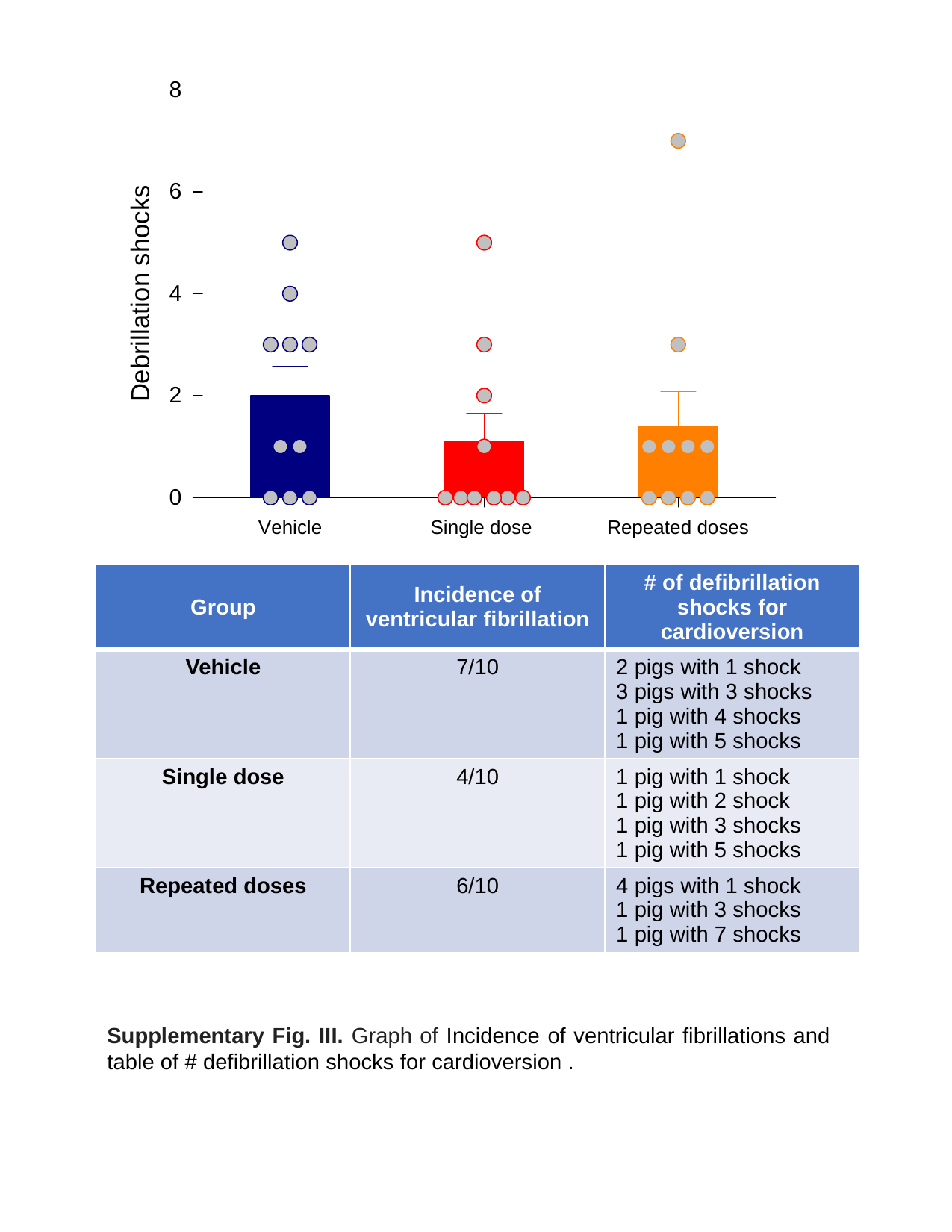

| Group | Incidence of ventricular fibrillation | # of defibrillation shocks for cardioversion |
| --- | --- | --- |
| Vehicle | 7/10 | 2 pigs with 1 shock 3 pigs with 3 shocks 1 pig with 4 shocks 1 pig with 5 shocks |
| Single dose | 4/10 | 1 pig with 1 shock 1 pig with 2 shock 1 pig with 3 shocks 1 pig with 5 shocks |
| Repeated doses | 6/10 | 4 pigs with 1 shock 1 pig with 3 shocks 1 pig with 7 shocks |
Supplementary Fig. III. Graph of Incidence of ventricular fibrillations and table of # defibrillation shocks for cardioversion .

## Slide 4
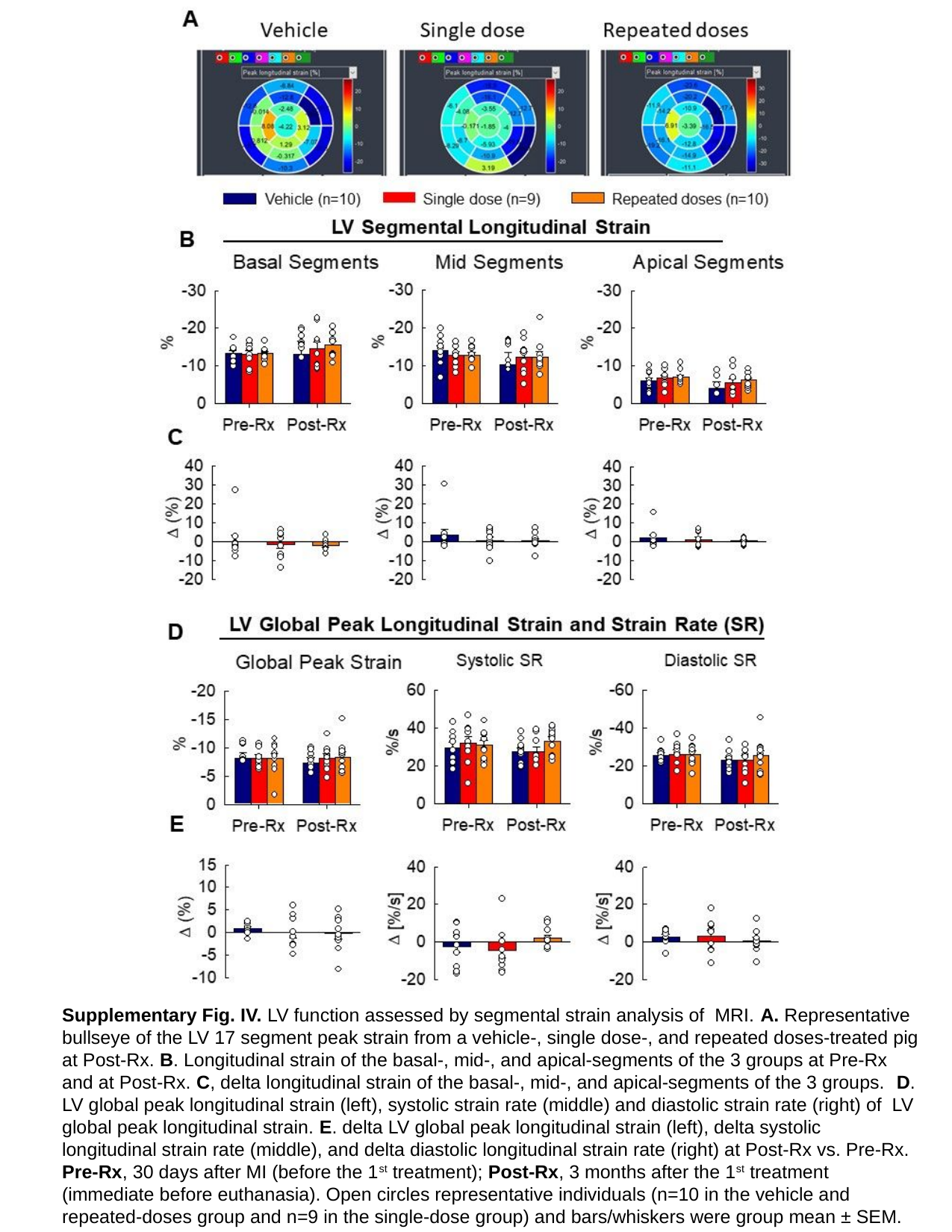

Supplementary Fig. IV. LV function assessed by segmental strain analysis of MRI. A. Representative bullseye of the LV 17 segment peak strain from a vehicle-, single dose-, and repeated doses-treated pig at Post-Rx. B. Longitudinal strain of the basal-, mid-, and apical-segments of the 3 groups at Pre-Rx and at Post-Rx. C, delta longitudinal strain of the basal-, mid-, and apical-segments of the 3 groups. D. LV global peak longitudinal strain (left), systolic strain rate (middle) and diastolic strain rate (right) of LV global peak longitudinal strain. E. delta LV global peak longitudinal strain (left), delta systolic longitudinal strain rate (middle), and delta diastolic longitudinal strain rate (right) at Post-Rx vs. Pre-Rx. Pre-Rx, 30 days after MI (before the 1st treatment); Post-Rx, 3 months after the 1st treatment (immediate before euthanasia). Open circles representative individuals (n=10 in the vehicle and repeated-doses group and n=9 in the single-dose group) and bars/whiskers were group mean ± SEM.

## Slide 5
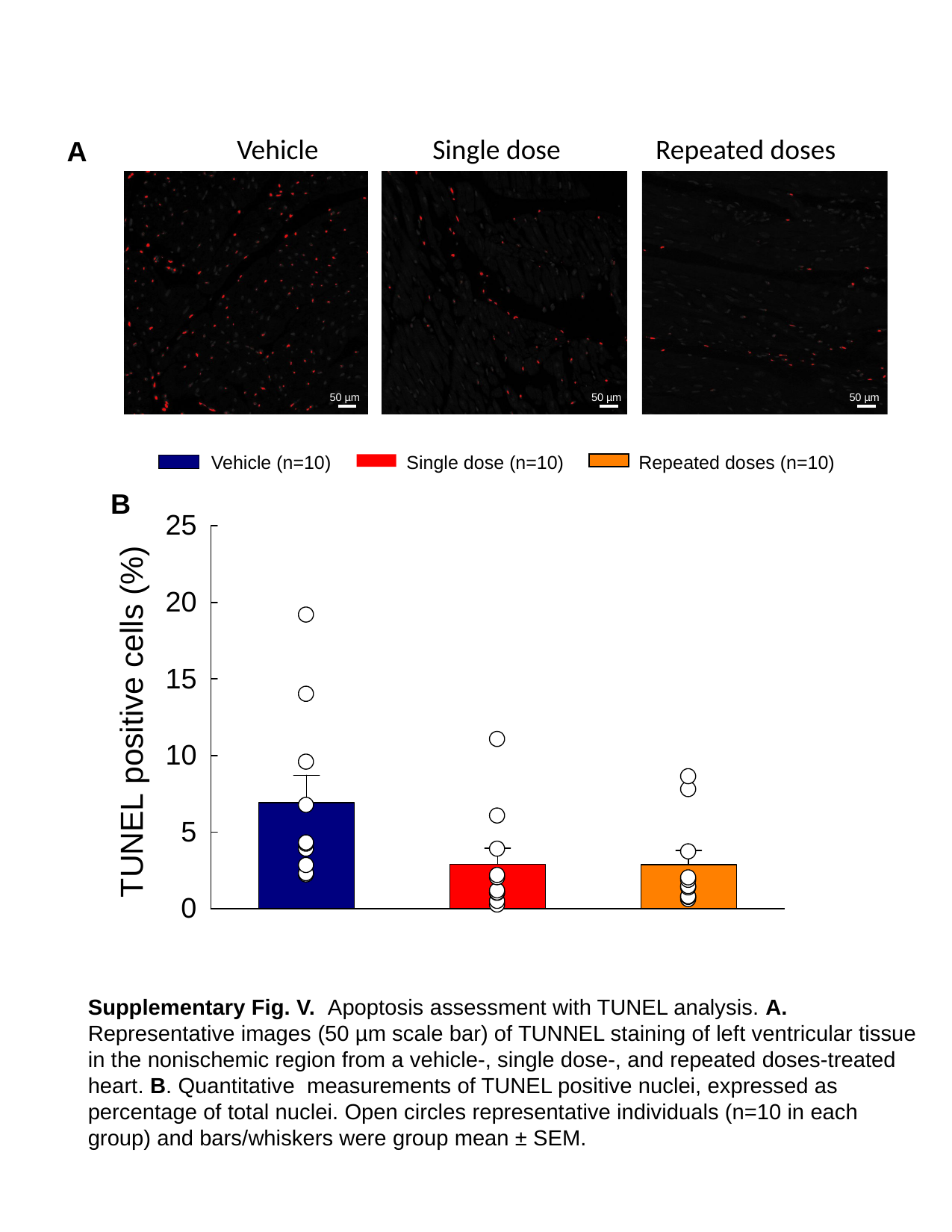

Vehicle Single dose Repeated doses
50 µm
50 µm
50 µm
A
Single dose (n=10)
Repeated doses (n=10)
Vehicle (n=10)
B
Supplementary Fig. V. Apoptosis assessment with TUNEL analysis. A. Representative images (50 µm scale bar) of TUNNEL staining of left ventricular tissue in the nonischemic region from a vehicle-, single dose-, and repeated doses-treated heart. B. Quantitative measurements of TUNEL positive nuclei, expressed as percentage of total nuclei. Open circles representative individuals (n=10 in each group) and bars/whiskers were group mean ± SEM.

## Slide 6
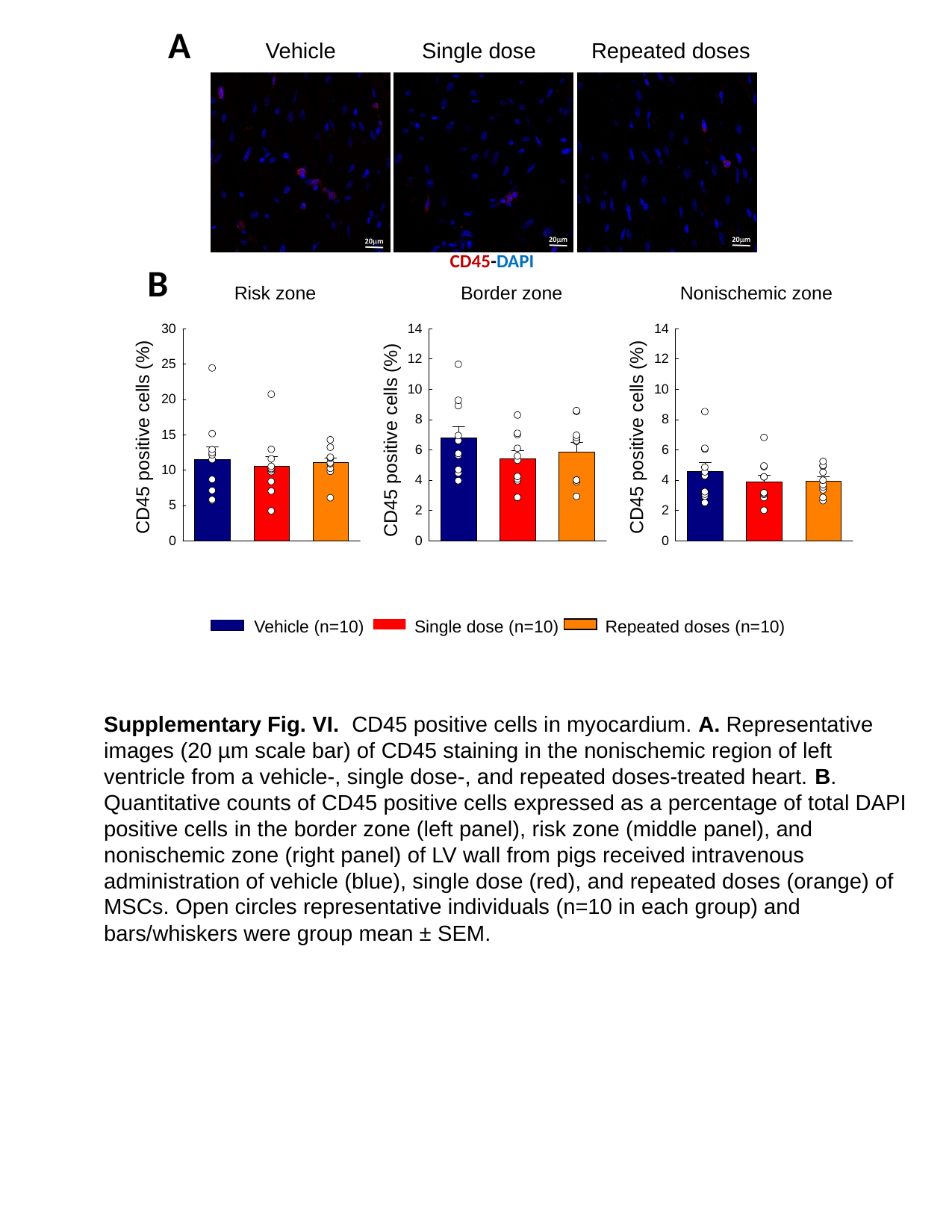

A
Vehicle Single dose Repeated doses
CD45-DAPI
B
Risk zone
CD45 positive cells (%)
Border zone
CD45 positive cells (%)
Nonischemic zone
CD45 positive cells (%)
Single dose (n=10)
Repeated doses (n=10)
Vehicle (n=10)
Supplementary Fig. VI. CD45 positive cells in myocardium. A. Representative images (20 µm scale bar) of CD45 staining in the nonischemic region of left ventricle from a vehicle-, single dose-, and repeated doses-treated heart. B. Quantitative counts of CD45 positive cells expressed as a percentage of total DAPI positive cells in the border zone (left panel), risk zone (middle panel), and nonischemic zone (right panel) of LV wall from pigs received intravenous administration of vehicle (blue), single dose (red), and repeated doses (orange) of MSCs. Open circles representative individuals (n=10 in each group) and bars/whiskers were group mean ± SEM.

## Slide 7
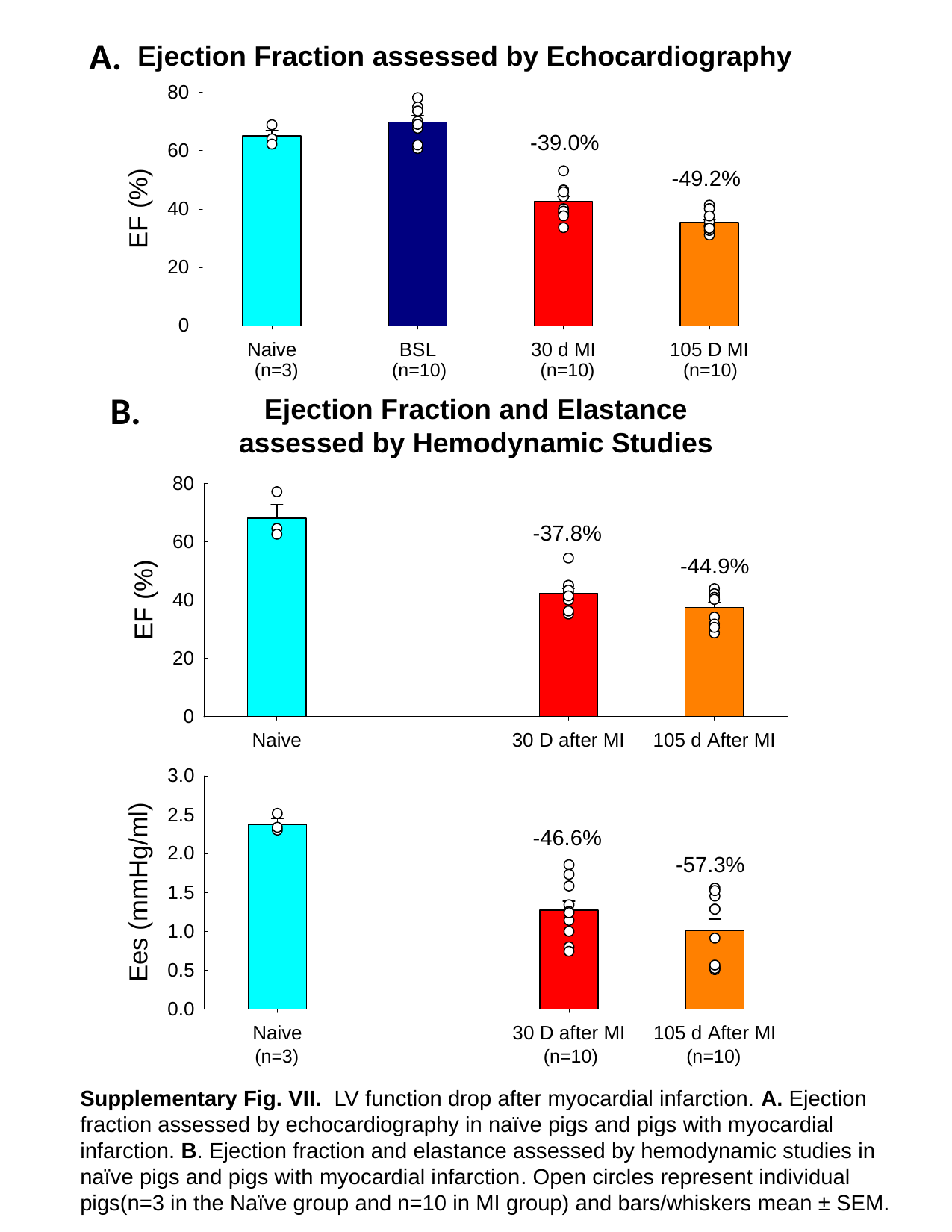

A.
-39.0%
-49.2%
(n=3) (n=10) (n=10) (n=10)
Ejection Fraction assessed by Echocardiography
B.
Ejection Fraction and Elastance assessed by Hemodynamic Studies
-37.8%
-44.9%
-46.6%
-57.3%
(n=3) (n=10) (n=10)
Supplementary Fig. VII. LV function drop after myocardial infarction. A. Ejection fraction assessed by echocardiography in naïve pigs and pigs with myocardial infarction. B. Ejection fraction and elastance assessed by hemodynamic studies in naïve pigs and pigs with myocardial infarction. Open circles represent individual pigs(n=3 in the Naïve group and n=10 in MI group) and bars/whiskers mean ± SEM.

## Slide 8
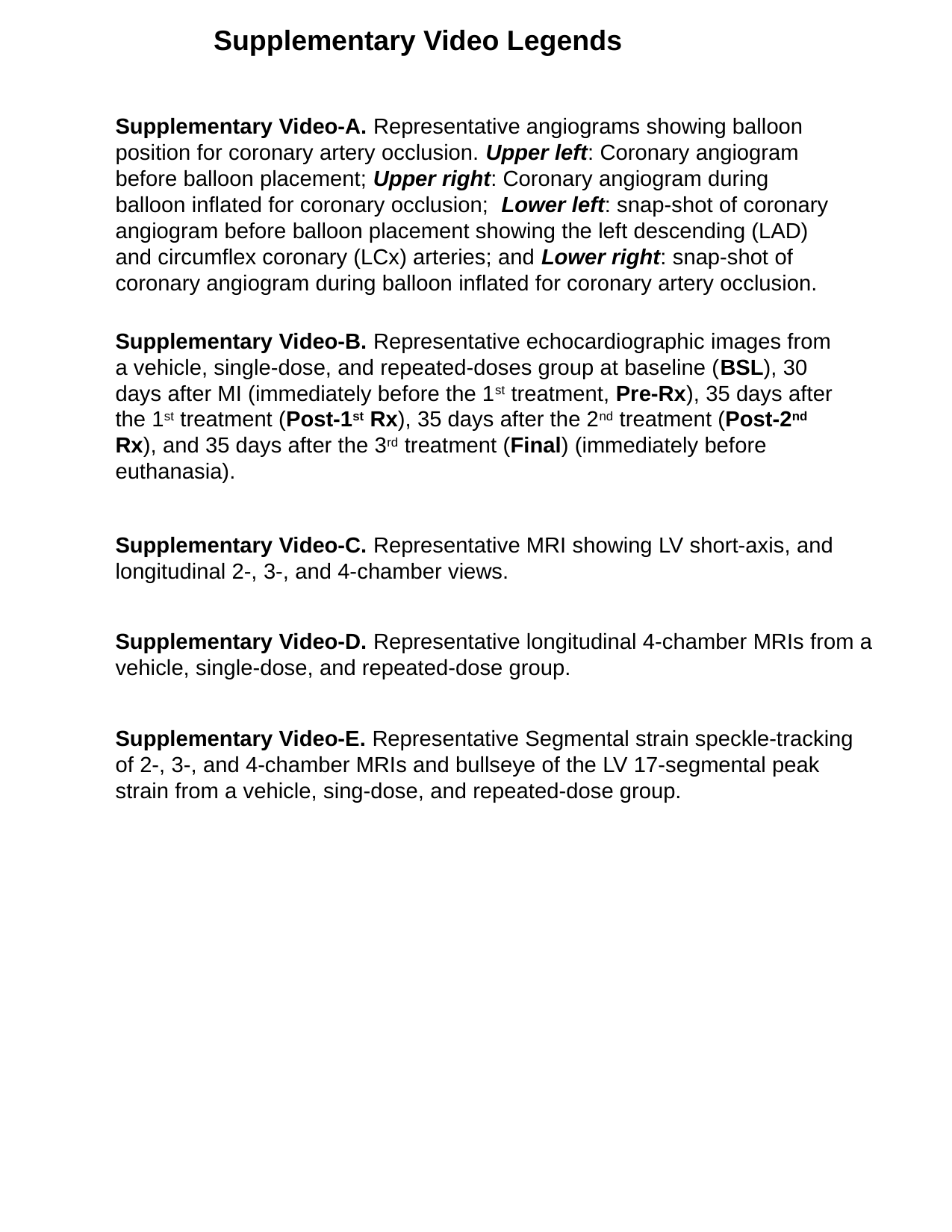

Supplementary Video Legends
Supplementary Video-A. Representative angiograms showing balloon position for coronary artery occlusion. Upper left: Coronary angiogram before balloon placement; Upper right: Coronary angiogram during balloon inflated for coronary occlusion; Lower left: snap-shot of coronary angiogram before balloon placement showing the left descending (LAD) and circumflex coronary (LCx) arteries; and Lower right: snap-shot of coronary angiogram during balloon inflated for coronary artery occlusion.
Supplementary Video-B. Representative echocardiographic images from a vehicle, single-dose, and repeated-doses group at baseline (BSL), 30 days after MI (immediately before the 1st treatment, Pre-Rx), 35 days after the 1st treatment (Post-1st Rx), 35 days after the 2nd treatment (Post-2nd Rx), and 35 days after the 3rd treatment (Final) (immediately before euthanasia).
Supplementary Video-C. Representative MRI showing LV short-axis, and longitudinal 2-, 3-, and 4-chamber views.
Supplementary Video-D. Representative longitudinal 4-chamber MRIs from a vehicle, single-dose, and repeated-dose group.
Supplementary Video-E. Representative Segmental strain speckle-tracking of 2-, 3-, and 4-chamber MRIs and bullseye of the LV 17-segmental peak strain from a vehicle, sing-dose, and repeated-dose group.
