# Supplementary material for: Intravenous infusions of mesenchymal stromal cells have cumulative beneficial effects in a porcine model of chronic ischaemic cardiomyopathy
Source: Cardiovasc Res. 2024 Aug 20;120(15):1939–52. doi: 10.1093/cvr/cvae173 (PMC11630033; doi:10.1093/cvr/cvae173)
Supplement: cvae173_Supplementary_Data [file cvae173_supplementary_data.zip › Supplementary Tables (06-26-24).docx]

**Supplementary Tables**

**Supplementary Table I:** Monoclonal antibodies

| **Antigen** | **Clone** | **Fluorochrome** | **Company** |
| --- | --- | --- | --- |
| CD90 | 5E10 | FITC | BioLegend |
| CD29 | NaM160-1A3 | Alexa Fluor 647 | BD Bioscience |
| CD105 | MEM-229 | FITC | Abcam |
| CD44 | MEM-263 | FITC | Abcam |
| CD31 | 390 | PE | eBioscience |
| CD45 | MIL-13 | FITC | ThermoScientific |

**Supplementary Table II:** Enrollment and Exclusions

One pig in the single dose group was unable to get the MRI scan and hemodynamic study at Pre-Rx and was excluded for MRI and hemodynamic data analysis.

| **Group** | **Initial Assigned** | **Exclusions** | | **Final Data Analysis** |
| --- | --- | --- | --- | --- |
|  |  | **During the study protocol** | **After the study protocol completed** |  |
| **Group I:**  **Vehicle control**  **(PBS, PBS, PBS)** | 13 | 3  (2 died of VF during occlusion; 1 euthanized due to severer breathing problem) | 0 | **10** |
| **Group II:**  **Single dose**  **(MSCs, PBS, PBS)** | 13 | 3  (1 died of VF during occlusion; 2 euthanized due to GI problem) | 0 | **10** |
| **Group III:**  **Repeated doses**  **(MSCs, MSCs, MSCs)** | 13 | 2  (1 died of VF 12 days after MI; 1 euthanized due to GI problem) | 1  (excluded due to high EF after MI) | **10** |
| **Total** | **39** | **9** | | **30** |

**Note:** One pig in the single dose group was unable to get the MRI scan and hemodynamic study at Pre-Rx and was excluded for MRI and hemodynamic data analysis.

**Supplementary Table III:** Age, body weight, heart weight, and days of treatment interval

|  | |  | **Vehicle**  **(n=10)** | | | **Single dose**  **(n=10)** | | | **Repeated doses**  **(n=10)** | | |
| --- | --- | --- | --- | --- | --- | --- | --- | --- | --- | --- | --- |
| **Age (days)** | | | 330.1 | ± | 19.1 | 331.5 | ± | 15.7 | 337.6 | ± | 15.7 |
| **Body weight**  **(kg)** | **BSL** | | 20.6 | ± | 1.2 | 20.5 | ± | 1.5 | 21.6 | ± | 1.7 |
|  | **Pre-Rx** | | 23.8 | ± | 1.5 | 23.3 | ± | 1.7 | 24.3 | ± | 1.9 |
|  | **Post-1^st^ Rx** | | 26.8 | ± | 1.6 | 26.0 | ± | 1.7 | 27.4 | ± | 2.0 |
|  | **Post-2^nd^ Rx** | | 30.2 | ± | 1.9 | 29.3 | ± | 2.0 | 30.8 | ± | 2.0 |
|  | **Post-3^rd^ Rx** | | 33.1 | ± | 1.9 | 33.5 | ± | 2.5 | 34.2 | ± | 2.1 |
| **Days of treatment interval** | **1^st^ Rx to 2^nd^ Rx** | | 34.8 | ± | 1.0 | 33.8 | ± | 0.9 | 36.0 | ± | 0.5 |
|  | **2^nd^ Rx to 3^rd^ Rx** | | 33.5 | ± | 1.1 | 31.7 | ± | 1.4 | 35.7 | ± | 0.6 |
|  | **3^rd^ Rx to Final** | | 33.4 | ± | 2.3 | 34.6 | ± | 1.4 | 36.6 | ± | 1.1 |
| **Heart weight (g)** | | | 158.5 | ± | 9.0 | 158.1 | ± | 13.2 | 158.2 | ± | 8.8 |
| **Spleen weight (g)** | | | 118.9 | ± | 17.4 | 149.9 | ± | 29.4 | 129.1 | ± | 24.3 |
| **Defibrillation shocks** | | | 2.0 | ± | 0.6 | 1.1 | ± | 0.5 | 1.4 | ± | 0.7 |

Data were mean±SEM

**Supplementary Table IV:**  Summary of echo data

|  | **Vehicle**  **(n=10)** | | | **Single dose**  **(n=10)** | | | **Repeated doses (n=10)** | | |
| --- | --- | --- | --- | --- | --- | --- | --- | --- | --- |
| **EF** | | | | | | | | | |
| **BSL** | 69.78 | ± | 2.23 | 66.00 | ± | 2.73 | 63.60 | ± | 2.10 |
| **Pre-Rx** | 42.56 | ± | 1.80***^b^*** | 39.57 | ± | 1.91***^b^*** | 38.34 | ± | 2.49***^b^*** |
| **Post-1^st^ Rx** | 38.50 | ± | 1.58 | 45.96 | ± | 1.54***** | 49.27 | ± | 1.59***^d^***** |
| **Post-2^nd^ Rx** | 36.64 | ± | 1.67 | 44.20 | ± | 1.88***** | 46.64 | ± | 1.19***^d^***** |
| **Post-3^rd^ Rx** | 35.42 | ± | 1.04***^d^*** | 42.79 | ± | 2.01***** | 46.16 | ± | 1.73***^d^***** |
|  |  |  |  |  |  |  |  |  |  |
| **EF ↓ at Pre-Rx from BSL** | -27.23 | ± | 2.14 | -26.43 | ± | 1.83 | -25.27 | ± | 2.71 |
| **Δ EF post 1^st^ Rx vs. Pre Rx** | -4.06 | ± | 0.99 | 6.38 | ± | 1.48****** | 10.94 | ± | 2.54****** |
| **Δ EF post 2^nd^ Rx vs. Pre Rx** | -5.92 | ± | 1.87 | 4.62 | ± | 1.54****** | 8.30 | ± | 2.88****** |
| **Δ EF post 3^rd^ Rx vs. Pre Rx** | -7.13 | ± | 1.63 | 3.21 | ± | 1.97****** | 7.83 | ± | 2.25****** |
| **EDV** | | | | | | | | | |
| **BSL** | 23.91 | ± | 1.66 | 25.31 | ± | 2.23 | 27.58 | ± | 2.48 |
| **Pre-Rx** | 43.81 | ± | 3.51***^b^*** | 41.02 | ± | 2.33***^b^*** | 40.98 | ± | 3.24***^b^*** |
| **Post-1^st^ Rx** | 62.40 | ± | 4.16***^d^*** | 57.73 | ± | 2.71***^d^****** | 46.78 | ± | 2.71***^c^**** |
| **Post-2^nd^ Rx** | 66.15 | ± | 6.99***^d^*** | 61.82 | ± | 2.93***^d^*** | 53.14 | ± | 4.11***^c^*** |
| **Post-3^rd^ Rx** | 62.97 | ± | 6.40***^d^*** | 53.71 | ± | 2.12***^d^*** | 57.58 | ± | 2.57***^c^*** |
| **Δ EDV at Pre-Rx from BSL** | 19.90 | ± | 3.77 | 15.70 | ± | 3.47 | 14.40 | ± | 2.67 |
| **Δ EDV post 1^st^ Rx vs. Pre Rx** | 17.99 | ± | 3.02 | 9.72 | ± | 1.63*** | 6.79 | ± | 2.51*** |
| **Δ EDV post 2^nd^ Rx vs. Pre Rx** | 18.35 | ± | 4.43 | 15.01 | ± | 1.58 | 10.15 | ± | 3.58 |
| **Δ EDV post 3^rd^ Rx vs. Pre Rx** | 19.17 | ± | 3.77 | 12.69 | ± | 2.44 | 10.60 | ± | 3.37 |
| **ESV** | | | | | | | | | |
| **BSL** | 7.24 | ± | 0.78 | 9.09 | ± | 0.98 | 10.29 | ± | 0.55 |
| **Pre-Rx** | 25.79 | ± | 2.38***^b^*** | 25.38 | ± | 1.70***^b^*** | 25.19 | ± | 2.37***^b^*** |
| **Post-1^st^ Rx** | 38.40 | ± | 3.90***^d^*** | 32.91 | ± | 1.17***^d^**** | 24.79 | ± | 1.86****** |
| **Post-2^nd^ Rx** | 42.86 | ± | 6.81***^d^*** | 34.97 | ± | 1.46***^d^*** | 28.50 | ± | 2.80***** |
| **Post-3^rd^ Rx** | 40.07 | ± | 4.26***^d^*** | 31.87 | ± | 1.67***^d^*** | 31.24 | ± | 3.47***^c^*** |
| **Δ ESV at Pre-Rx from BSL** | 18.06 | ± | 2.29 | 16.29 | ± | 1.81 | 15.60 | ± | 2.25 |
| **Δ ESV post 1^st^ Rx vs. Pre Rx** | 15.61 | ± | 3.23 | 5.53 | ± | 1.69***** | -0.40 | ± | 1.84****** |
| **Δ ESV post 2^nd^ Rx vs. Pre Rx** | 19.07 | ± | 5.70 | 10.60 | ± | 1.30 | 3.31 | ± | 2.36***** |
| **Δ ESV post 3^rd^ Rx vs. Pre Rx** | 16.28 | ± | 3.32 | 7.30 | ± | 1.43***** | 6.05 | ± | 2.36***** |
| **SV** | | | | | | | | | |
| **BSL** | 16.67 | ± | 1.33 | 16.21 | ± | 1.56 | 17.35 | ± | 1.79 |
| **Pre-Rx** | 17.95 | ± | 1.41 | 15.64 | ± | 1.03 | 15.90 | ± | 1.71 |
| **Post-1^st^ Rx** | 23.99 | ± | 2.40 | 24.87 | ± | 1.48***^d^*** | 22.28 | ± | 1.45***^c^*** |
| **Post-2^nd^ Rx** | 23.29 | ± | 2.83 | 26.85 | ± | 2.28***^d^*** | 24.94 | ± | 1.81***^d^*** |
| **Post-3^rd^ Rx** | 22.72 | ± | 2.00 | 21.83 | ± | 1.22***^c^*** | 26.34 | ± | 2.19***^d^*** |
| **Δ SV at Pre-Rx from BSL** | 13.96 | ± | 12.67 | 9.74 | ± | 18.04 | -6.67 | ± | 7.06 |
| **Δ SV post 1^st^ Rx vs. Pre Rx** | 33.61 | ± | 7.58 | 62.15 | ± | 9.61***** | 55.45 | ± | 18.04 |
| **Δ SV post 2^nd^ Rx vs. Pre Rx** | 28.60 | ± | 9.92 | 72.68 | ± | 11.72***** | 72.81 | ± | 20.27 |
| **Δ SV post 3^rd^ Rx vs. Pre Rx** | 30.88 | ± | 12.07 | 44.80 | ± | 11.13 | 81.72 | ± | 22.72 |

Data were mean±SEM

***^a^*** *P*<0.05, ***^b^*** *P*<0.01 vs. BSL; ***^C^*** *P*<0.05, ***^d^*** *P*<0.01 vs. Pre-Rx; ******* *P*<0.05, ******** *P*<0.01 vs. vehicle

*P* values were calculated by two-way repeated-measures ANOVA with Bonferroni t-test.

**Supplementary Table V:**  Summary of MRI data

|  | **Vehicle**  **(n=10)** | | | **Single dose**  **(n=9)** | | | **Repeated doses**  **(n=10)** | | |
| --- | --- | --- | --- | --- | --- | --- | --- | --- | --- |
| **Pre-Rx** | | | | | | | | | |
| **EF (%)** | 39.99 | ± | 1.31 | 38.18 | ± | 1.69 | 38.90 | ± | 1.29 |
| **EDV (ml)** | 45.05 | ± | 4.51 | 48.11 | ± | 4.06 | 43.20 | ± | 2.86 |
| **ESV (ml)** | 27.24 | ± | 3.07 | 29.75 | ± | 2.64 | 26.46 | ± | 1.97 |
| **SV (ml)** | 17.81 | ± | 1.56 | 18.36 | ± | 1.70 | 16.74 | ± | 1.14 |
| **Post-Rx** | | | | | | | | | |
| **EF (%)** | 35.89 | ± | 2.26***a*** | 38.28 | ± | 1.89 | 43.45 | ± | 2.11***a**** |
| **EDV (ml)** | 64.33 | ± | 4.77***b*** | 59.88 | ± | 4.16***a*** | 60.82 | ± | 5.02***b*** |
| **ESV (ml)** | 41.30 | ± | 4.52***b*** | 36.92 | ± | 2.66***a*** | 34.75 | ± | 3.51***b*** |
| **SV (ml)** | 23.03 | ± | 1.65 | 22.81 | ± | 1.91 | 25.87 | ± | 2.11***b*** |
| **Δ at Post-Rx vs. Pre-Rx** | | | | | | | | | |
| **Δ EF (%)** | -3.40 | ± | 1.95 | 0.11 | ± | 2.07 | 4.56 | ± | 1.68** |
| **Δ EDV (ml)** | 19.28 | ± | 2.83 | 10.26 | ± | 3.59 | 17.62 | ± | 4.39 |
| **Δ ESV (ml)** | 14.06 | ± | 2.90 | 6.16 | ± | 2.07 | 8.29 | ± | 3.02 |
| **Δ SV (ml)** | 5.22 | ± | 1.76 | 3.93 | ± | 2.04 | 9.13 | ± | 1.83 |

Data were mean±SEM

***^a^*** *P*<0.05, ***^b^*** *P*<0.01 vs. Pre-Rx; ******* *P*<0.05, ******** *P*<0.01 vs. vehicle

*P* values were calculated by two-way repeated-measures ANOVA with Bonferroni t-test.

**Supplementary Table VI-a:**  LV segmental strain


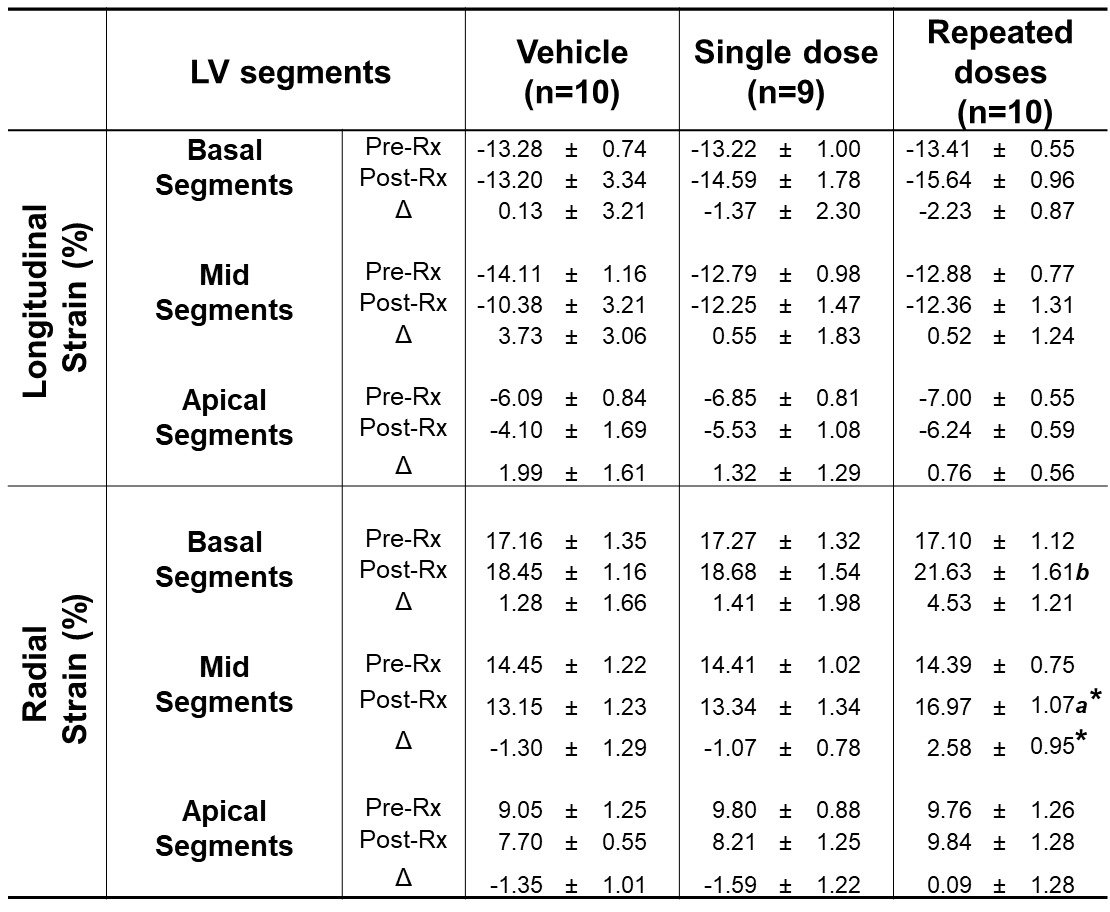


Data were mean±SEM

***^a^*** *P*<0.05, ***^b^*** *P*<0.01 vs. Pre-Rx; ******* *P*<0.05 vs. vehicle

*P* values were calculated by two-way repeated-measures ANOVA with Bonferroni t-test.

LV segments

basal anterior, basal anteroseptal, basal inferoseptal, basal inferior, basal inferolateral, and basal anterolateral

mid anterior, mid anteroseptal, mid inferoseptal, mid inferior, mid inferolateral, and mid anterolateral

apical anterior, apical septal, apical inferior, apical lateral, and apex

Basal:

Mid:

Apical:

**Supplementary Table VI-b:**  LV Global Peak Strain and Strain Rate (SR)


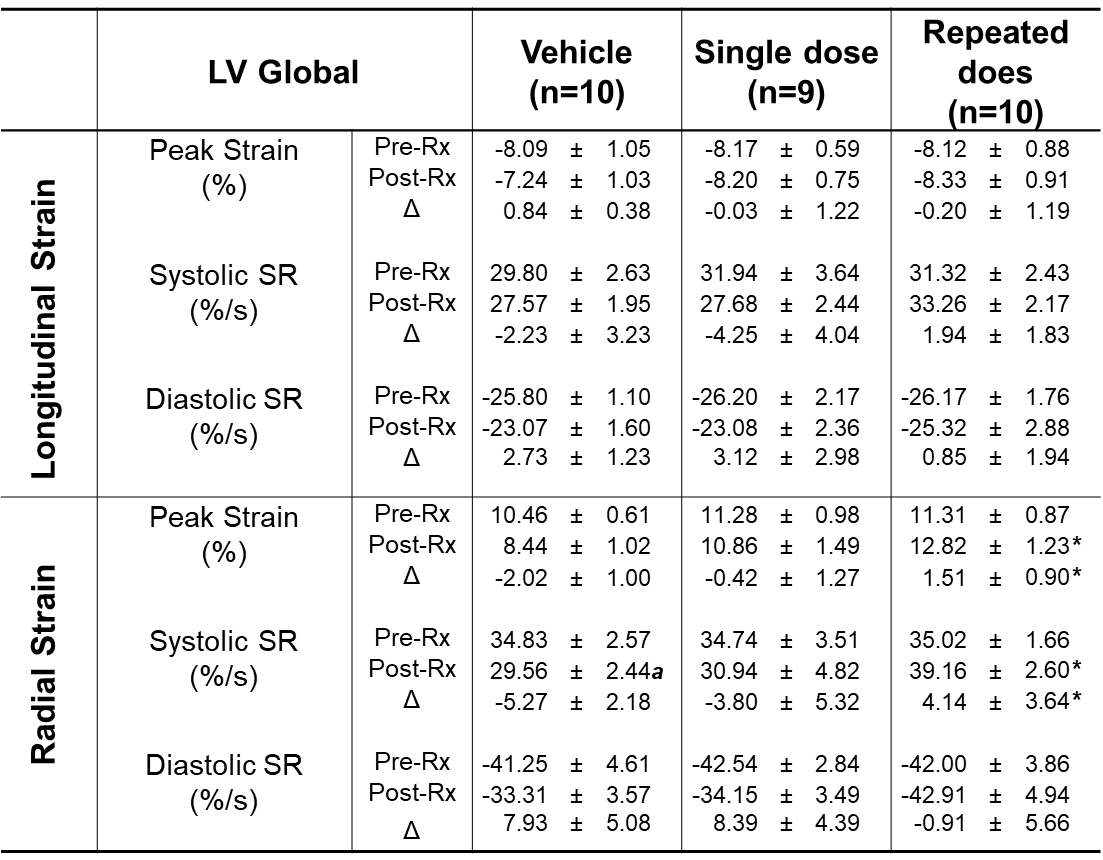


Data were mean±SEM

***^a^*** *P*<0.0z5, ***^b^*** *P*<0.01 vs. Pre-Rx; ******* *P*<0.05 vs. vehicle

*P* values were calculated by two-way repeated-measures ANOVA with Bonferroni t-test.

**Supplementary Table VII:**  Hemodynamic data


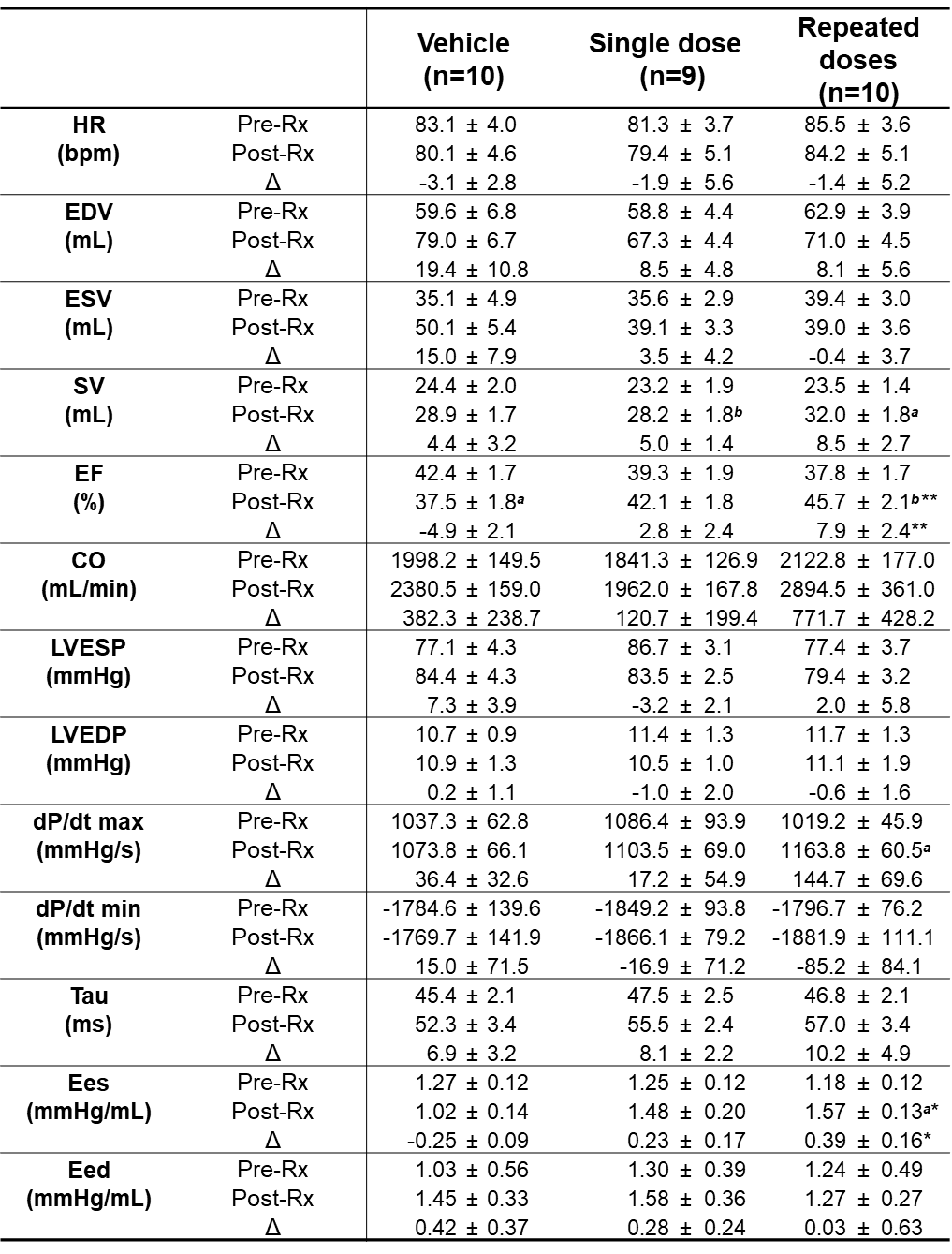


Data were mean±SEM ***Ees***, end-systolic elastance; ***Eed***, end-diastolic elastance

***^a^*** *P*<0.05, ***^b^*** *P*<0.01 vs. Pre-Rx; ******* *P*<0.05, ******** *P*<0.01 vs. vehicle.

*P* values were calculated by two-way repeated-measures ANOVA with Bonferroni t-test.

**Supplementary Table VIII:**  Capillary Density (#/mm^2^)


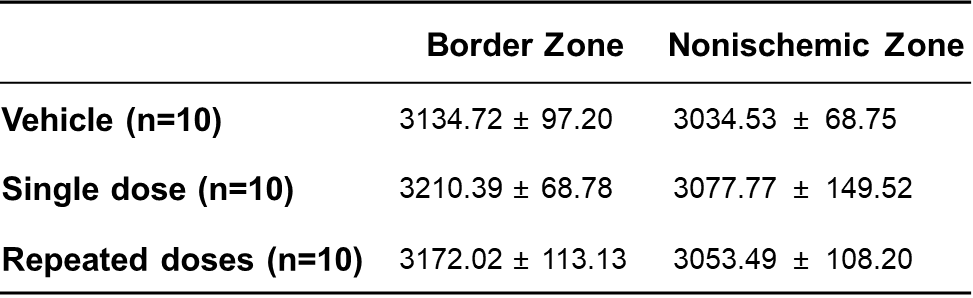


Data were mean±SEM
